# 5-Azacytidine modulates interferon regulatory factor 1 in macrophages to exert a cardioprotective effect

**DOI:** 10.1038/srep15768

**Published:** 2015-10-29

**Authors:** Hye-yun Jeong, Wan Seok Kang, Moon Hwa Hong, Hae Chang Jeong, Myun-Geun Shin, Myung Ho Jeong, Yong Sook Kim, Youngkeun Ahn

**Affiliations:** 1 Research Laboratory of Cardiovascular Regeneration, Chonnam National University Hospital, Gwangju, Republic of Korea; 2 Department of Molecular Medicine, Graduate School, Chonnam National University, Gwangju, Republic of Korea; 3 Department of Cardiology, Chonnam National University Hospital, Gwangju, Republic of Korea; 4 Department of Laboratory Medicine, Chonnam National University Hwasun Hospital, Hwasun, Republic of Korea; 5 Biomedical Research Center, Chonnam National University Hospital, Gwangju, Republic of Korea

## Abstract

Macrophages are actively involved in inflammatory responses during the progression of cardiac injury, including myocardial infarction (MI). A previous study showed that 5-azacytidine (5AZ), a DNA methylation inhibitor, can ameliorate cardiac injury by shifting macrophages toward an anti-inflammatory phenotype via iNOS inhibition. Here, we show that the beneficial effect of 5AZ is associated with sumoylation of interferon regulatory factor-1 (IRF1) in macrophages. IRF1 is a critical transcription factor for iNOS induction and is antagonized by IRF2. In the stimulated macrophages, IRF1 accumulated in the nucleus without degradation by 5AZ treatment. In animal study, 5AZ administration resulted in significant improvements in cardiac function and fibrosis. IRF1-expressing macrophages were more abundant in the 5AZ-treated MI group than in the PBS-treated MI group. Because sumoylated IRF1 is known to mimic IRF2, we examined the IRF1 sumoylation. Sumoylated IRF1 was resistant to degradation and significantly increased in the 5AZ-treated MI group. Collectively, 5AZ had a protective effect after MI by potentiation of IRF1 sumoylation and is suggested as a novel therapeutic intervention for cardiac repair.

Myocardial infarction (MI) is a main cause of death worldwide despite the rapid development of therapeutic modalities and drugs. In response to the loss of cardiomyocytes, myofibroblasts are activated to form mature scars, and monocytes infiltrate the lesion. In response to the regional inflammation due to cardiac injury, circulating monocytes are recruited into damaged tissue and differentiate into macrophages^1^. The infiltrated macrophages actively facilitate the removal of pathogens, tissue healing, and resolution of inflammation, whereas uncontrolled inflammation leads to cardiac remodeling and dysfunction.

Macrophages comprise two phenotypically distinct subsets depending on their unique roles: the M1 type and the M2 type. Classically activated M1 macrophages are associated with pro-inflammatory mediators such as inducible nitric oxide synthase (iNOS)-derived NO, tumor necrosis factor-α (TNF-α), and interleukin-12 (IL-12). Alternatively, activated M2 macrophages express IL-10, Ym1 (chitinase 3-like 3), and Fizz1 (resistin like beta); upregulate arginase-1 (Arg1) and CD206; and support wound healing. In macrophages, iNOS is highly inducible by cytokines such as lipopolysaccharide (LPS) and interferon-γ (IFN-γ)^2^,^3^. These specialized phenotypes of monocytes and macrophages are critical drivers in the pathophysiology of cardiovascular diseases^4^.

We have previously reported in MI rat model that 5-azacytidine (5AZ) exerts its cardioprotective effect by moving macrophages toward the alternative phenotype in MI^5^. iNOS was suggested as a target of 5AZ in activated macrophages; however, the specific factor in macrophages responsible for 5AZ was unclear.

In the present study, we identified interferon regulatory factor-1 (IRF1) as one of the factors capable of affecting iNOS expression in activated macrophages. IRF1, which has been identified as a transcription factor responsible for iNOS induction, is expressed in macrophages by extracellular stress-induced stimulations^6^,^7^. The IRF family, which comprises 9 members in mammalian cells, is implicated in host defense, cell growth, and immune regulation^8^,^9^,^10^. The transcriptional activity of IRF1 is regulated by stimuli such as viral infection and inflammatory cytokines; transcription of IRF1 results in tumor suppression, apoptosis, immune responses, and cell growth^11^,^12^,^13^.

Despite the fact that infiltrated macrophages play an important role in cardiovascular disease, little is known about the underlying mechanism of 5AZ on macrophages to accommodate their pathophysiologic phenotype. To dissect the role of 5AZ-associated macrophage polarization, we focused on the modulation of IRF1. In the present study, we show that the pro-inflammatory polarization of macrophages in myocardial infarction is associated with IRF1 modulation and identify 5AZ as a potential pharmacological therapy to protect cardiac function.

## Results

### 5AZ differentially regulates iNOS and IRF1 in response to LPS stimulation

**Figure 1:**
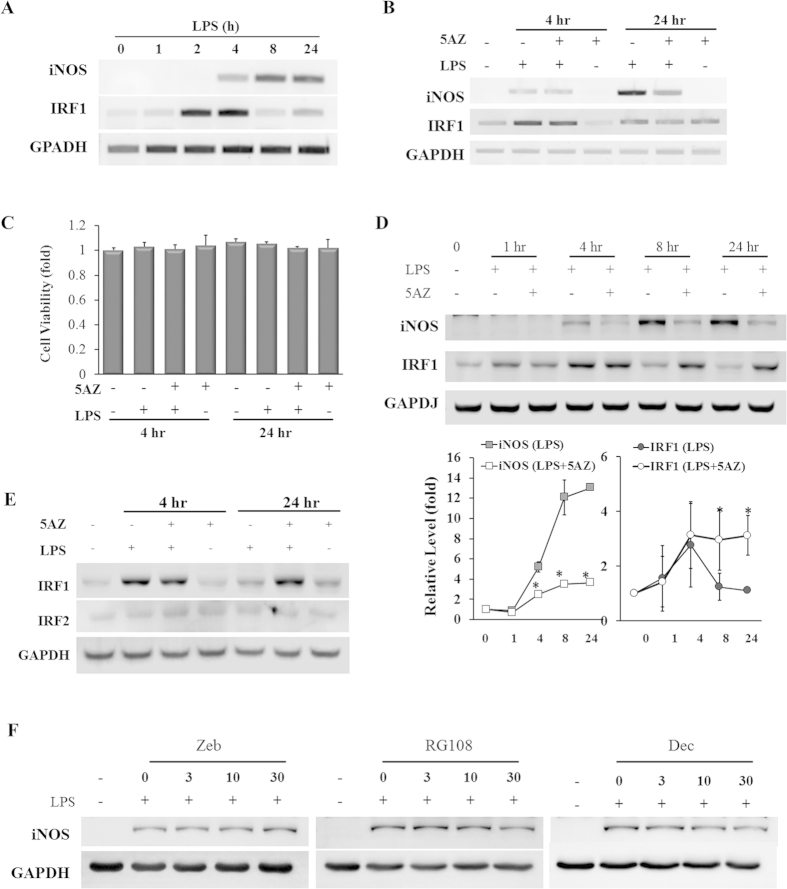
5AZ regulates iNOS and IRF1 differentially in LPS-activated RAW264.7 cells. (**A**) Induction patterns of iNOS and IFR1 in response to LPS stimulation were determined by RT-PCR. RAW264.7 cells were treated with LPS for the indicated time, and mRNA levels of iNOS and IRF1 were monitored. (**B**) Effect of 5AZ on mRNA levels of iNOS and IRF1. Cells were treated with LPS in the absence or presence of 5AZ for 4 hours or 24 hours. (**C**) Cell viability was assessed by MTT assay and was not influenced by LPS and 5AZ treatment. (**D**) Protein expression patterns of iNOS and IRF1 in response to LPS stimulation were determined by Western blot. Cells were treated with LPS in the absence or presence of 5AZ for the indicated time. Results expressed as mean ± SD. *Significantly different from cells treated with LPS alone, p < 0.05. (**E**) Unlike that of IRF1, the protein level of IRF2 was not induced by LPS treatment. (**F**) The effects of DNA methyltransferase inhibitors on iNOS protein induction in LPS-stimulated RAW264.7 cells. Cells were stimulated with LPS for 24 hours in the presence of zebularin (Zeb), RG108, and decitabine (Dec) with various concentrations. iNOS protein expression was analyzed by immunoblot.

To examine the kinetics of iNOS and IRF1 expression in response to LPS, cells were treated with LPS for various time intervals. LPS induced iNOS mRNA in a time-dependent manner. On the other hand, IRF1 mRNA was significantly increased at 2 to 4 hours of LPS stimulation but then rapidly decreased to basal levels (Fig. 1A). In the presence of 5AZ, iNOS mRNA was not induced by LPS for 24 hours. By contrast, the levels of IRF1 mRNA were not influenced by the presence of 5AZ (Fig. 1B). Cell viability was assessed by MTT assay and 5AZ did not exert any toxic effect (Fig. 1C).

We next examined the protein levels of iNOS and IRF1 in LPS-stimulated RAW264.7 cells. iNOS protein was induced in a time-dependent manner, and IRF1 protein peaked at 4 hours with sequential degradation within 24 hours. In the presence of 5AZ, iNOS protein was not induced by LPS, whereas IRF1 was induced normally and peaked at 4 hours without sequential degradation (Fig. 1D). The protein levels at each time point were quantified and expressed as graphs to show that 5AZ altered the natural kinetics of iNOS and IRF1. IRF2 is known to act as an antagonist of IRF1^14^, and thus we examined whether IRF2 was also affected by 5AZ. IRF2 protein was neither induced by LPS nor changed by 5AZ treatment (Fig. 1E). This result indicated that the inhibitory effect of 5AZ on iNOS was not mediated by IRF2 induction in stimulated macrophages.

To examine whether the inhibitory effect on iNOS induction is unique to 5AZ, we tested other DNA methyltransferase inhibitors such as zebularine (Zeb), RG108, and decitabine (Dec). Unlike to 5AZ, these compounds did not show a significant inhibition of LPS-induced iNOS protein induction (Fig. 1F).

5AZ reduced NO generation to 56.44 ± 4.20% of that in cells treated with LPS alone (Supplementary Fig. S1A). LPS-stimulated iNOS promoter activity was also reduced in the presence of 5AZ (Supplementary Fig. S1B). The relative fold-increase values were 181.80 ± 12.28 in LPS-treated and 95.35 ± 43.84 in LPS- and 5AZ-treated cells at 4 hours and 255.27 ± 41.86 in LPS-treated and 63.54 ± 27.20 in LPS- and 5AZ-treated cells at 24 hours. Next we examined whether NF-κB is activated by LPS and is involved in the inhibitory action of 5AZ. RAW264.7 cells were treated with LPS and the protein level of IκBα was investigated by Western blot. IκBα was rapidly reduced in LPS-stimulated cells, and this decrease was not affected by 5AZ (Supplementary Fig. S1C). Nuclear translocation of p65 and its phosphorylation were increased in LPS-stimulated cells regardless of 5AZ treatment (Supplementary Fig. S1D). In addition to p65, both p50 and p52 also accumulated in the nuclear fraction in LPS-stimulated cells, and this was not affected by 5AZ treatment.

### IRF1 is involved in LPS-stimulated iNOS induction

**Figure 2:**
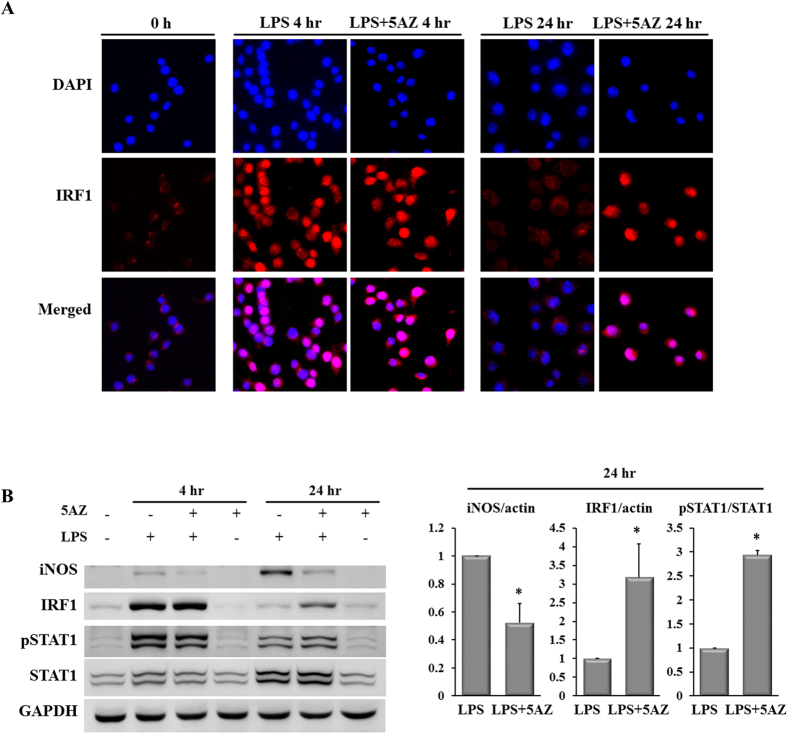
5AZ upregulates IRF1 in LPS-activated RAW264.7 cells. (**A**) Immunofluorescence of IRF1 in intact unstimulated and LPS-stimulated RAW264.7 cells was examined. IRF1 accumulated in the nucleus at 4 hours of LPS stimulation and then returned to the basal state at 24 hours. On the other hand, IRF1 stayed in the nucleus at 24 hours of stimulation with LPS and 5AZ. The nuclei were readily identified with DAPI. (**B**) Effect of 5AZ on the protein expressions of iNOS, IRF1, and STAT1. Cells were treated with LPS in the absence or presence of 5AZ for 4 hours or 24 hours. Results expressed as mean ± SD. *Significantly different from cells treated with LPS alone, p < 0.05.

Because LPS-induced IRF1 remained highly expressed in the immunoblotting analyses, its intracellular localization was examined by immunocytochemical staining. In non-stimulated cells, few IRF1-positive cells were observed. At 4 hours of LPS stimulation, the quantity of IRF1 was increased in nuclei in both in LPS- and LPS+5AZ-treated cells. At 24 hours, most of the nuclear IRF1 had disappeared in LPS-treated cells, whereas nuclear IRF1 remained upregulated in LPS+5AZ-treated cells (Fig. 2A). These observations correlated with the results from RT-PCR and Western blot.

STAT1 is a transcriptional factor for IRF1 induction, and is activated by phosphorylation on its serine residue. We assessed the phosphorylation of STAT1 to examine whether STAT1 was involved in the effect of 5AZ on IRF1 expression in RAW264.7 cells. STAT1 was phosphorylated to be active at 4 hours and dephosphorylated at 24 hours of LPS stimulation. In the presence of 5AZ, the level of phosphorylated STAT1 was higher than in cells treated with LPS alone. In cells treated with both LPS and 5AZ for 24 hours, iNOS was not induced, IRF1 remained upregulated, and phosphorylated STAT1 was increased compared with LPS-treated cells (Fig. 2B).

### Degradation of IRF1 is partially inhibited by 5AZ treatment

**Figure 3:**
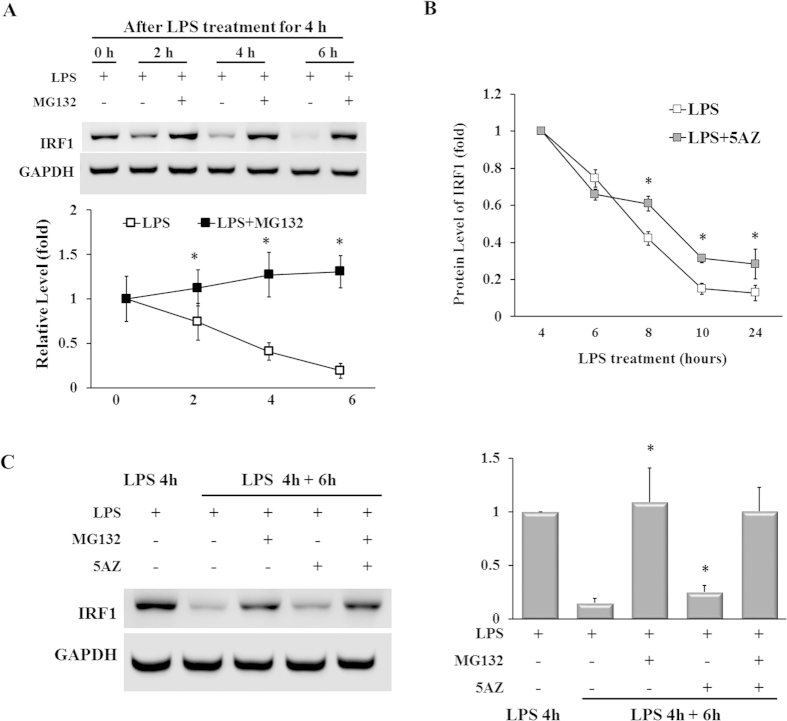
5AZ increases the stability of IRF1 protein. (**A**) Cells were stimulated with LPS for 4 hours followed by further treatment with MG132 and were harvested at the times shown. The immunoblots show the levels of IRF1 detected by using the IRF1 antibody; the data were analyzed by densitometry. As shown, upregulated IRF1 was rapidly decreased over time, whereas it remained highly expressed in the presence of MG132, a proteasome inhibitor. Results expressed as mean ± SD. *p < 0.05. (**B**) Cells were treated with LPS for 4 hours and additionally incubated at various time intervals to analyze IRF1 by immunoblot. After 4 hours, IRF1 protein was rapidly reduced, whereas the decrease was attenuated by 5AZ treatment. Results expressed as mean ± SD. *p < 0.05. (**C**) Cells were treated with LPS for 4 hours and further treated with LPS, MG132, or 5AZ as indicated for another 6 hours. IRF1 protein was dramatically decreased by LPS treatment for 10 hours, whereas MG132 almost completely blocked the degradation of IRF1. 5AZ treatment retarded the IRF1 degradation with statistical significance. Results expressed as mean ± SD. *Significantly different from cells treated with LPS for 10 hours, p < 0.05.

Because induced IRF1 protein was subsequently decreased within 24 hours in LPS-stimulated RAW264.7 cells, we examined the involvement of proteasomal degradation by using MG132, a proteosome inhibitor. RAW264.7 cells activated with LPS for 4 hours were incubated with fresh medium with or without MG132 (10 μM), and at various time intervals cell lysates were assayed by immunoblotting for IRF1. The rapid decrease of IRF1 protein by 0.19 ± 0.08 fold after 6 hours was completely blocked by the proteasome inhibitor MG132. In the presence of MG132, the induced IRF1 protein was preserved over time (Fig. 3A).

To assess the effect of 5AZ on IRF1 protein stability, RAW 264.7 cells were activated for 4 hours with LPS and then incubated in the presence or absence of 5AZ. The protein level of IRF1 peaked at 4 hours and then rapidly decreased. The relative IRF1 level was 0.75 ± 0.05 fold at 6 hours, 0.42 ± 0.04 fold at 8 hours, 0.15 ± 0.03 fold at 10 hours, and 0.13 ± 0.04 fold at 24 hours in cells stimulated with LPS alone. In the presence of 5AZ, the IRF1 protein levels were 0.66 ± 0.03 fold at 6 hours, 0.61 ± 0.04 fold at 8 hours, 0.31 ± 0.02 fold at 10 hours, and 0.28 ± 0.08 fold at 24 hours (Fig. 3B).

Next, we compared the effect of 5AZ with that of MG132, an inhibitor of protein degradation. After induction of IRF1 with LPS for 4 hours, further incubation for 6 hours with combinational treatment followed. LPS-induced IRF1 protein was maintained by either MG132 treatment (1.09 ± 0.32 fold) or MG132+5AZ treatment (1.06 ± 0.22 fold, Fig. 3C). Thus, the kinetics of IRF1 protein was regulated by the balance between degradation and synthesis of IRF1 protein, and 5AZ may contribute to a partial inhibition of degradation of IRF1.

### 5AZ administration in an MI model preserves cardiac function through phenotype modulation of the infiltrated macrophages in the myocardium

**Figure 4:**
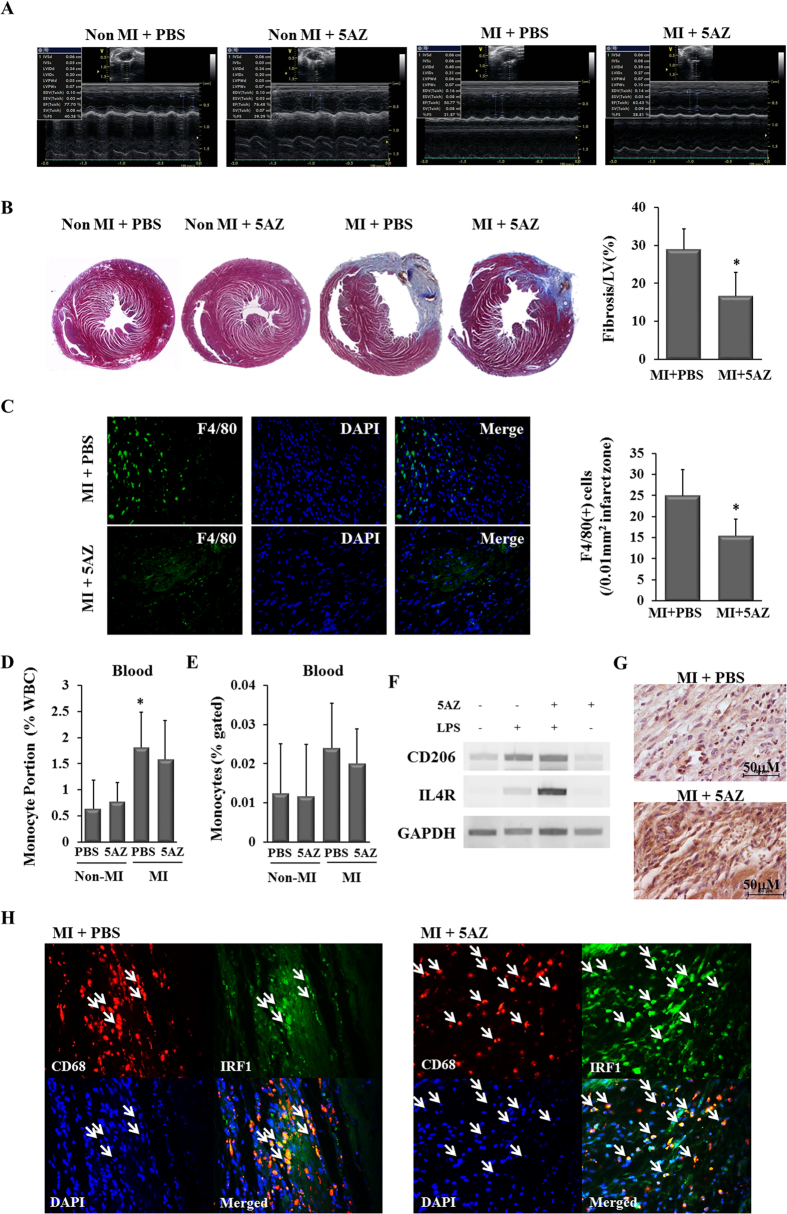
5AZ improves LV function and regulated the phenotype of infiltrated macrophages. (**A**) Representative echocardiograms showed more preserved cardiac function in the MI + 5AZ group than in the MI + PBS group at 2 weeks after MI. (**B**) Cardiac fibrosis was examined by Masson’s trichrome staining and quantified. *Fibrotic area was significantly smaller in the MI + 5AZ group than in the MI+PBS group, p < 0.05. (**C**) The representative images of infiltrated F4/80(+) macrophages in the infarcted myocardium were shown and the number of macrophages was expressed in a graph (n = 4 per group). (**D**) Circulating monocytes were counted by complete blood count and the percent of WBC was expressed. *The portion of circulating monocyte was significant higher than other three groups, p < 0.05 (**E**) CD45(+)CD14(+) monocytes from whole blood were quantified by flow cytometry and the percent of monocytes of CD45(+) cells was expressed. (**F**) The mRNA levels of CD206 and IL4R, M2 macrophage markers, were potentiated by 5AZ in LPS-stimulated RAW264.7 cells. (**G**) Immunohistochemical staining for the anti-inflammatory M2 macrophage marker CD206 in mouse infracted myocardium. CD206(+) cells were more frequently observed in the MI+5AZ group than in MI + PBS group. (**H**) IRF1-expressing macrophages in the infarcted myocardium. Double immunohistochemical staining for identification of IFR-1 expression in infiltrated macrophages in rat infarcted myocardium. CD68(+) IRF1(+) cells were abundant in the MI+ 5AZ group compared with the MI+PBS group. White arrows indicate the CD68(+) IRF1(+) cells.

The effect of 5AZ on post-MI cardiac function was assessed by echocardiography. As shown in a representative echocardiogram and by functional indexes, cardiac contractile function was preserved by 5AZ administration (Fig. 4A, Table 1). Left ventricular ejection fraction and fractional shortening were significantly preserved in the 5AZ-treated MI group. On the other hand, the morphometric indexes did not differ significantly between the MI + PBS group and the MI+5AZ group.

Cardiac fibrosis was analyzed by Masson’s trichrome staining and quantified. The percentage of left ventricular fibrotic area was smaller in the MI + 5AZ group than in the MI+PBS group (28.85 ± 5.44% vs. 16.58 ± 6.30%, *p* < 0.05, Fig. 4B).

To compare the number of infiltrated macrophages into infarcted myocardium between two groups, we quantified F4/80 (+) cells. The macrophages were more abundant in the MI+PBS group than in the MI+5AZ group (Fig. 4C). Next, blood monocytes were quantified to examine whether they were reduced by 5AZ treatment. Circulating monocytes were identified by Wright staining (Fig. S3A) and the number of monocytes were calculated by complete blood count (CBC) and flow cytometry (Fig. S3B). In CBC counts, the portion of monocytes (%) in the MI+PBS group was statistically higher than other three groups (Fig. 4D). On the other hand, there were no statistical differences between the groups in the flow cytometric results. Importantly, there was no significant reduction in blood monocytes by 5AZ treatment (Fig. 4E).

Because 5AZ significantly reduced the expression and activity of the proinflammatory M1 marker iNOS, we examined the effect of 5AZ on the antiinflammatory M2 markers. In LPS-treated RAW264.7 cells, 5AZ potentiated M2 phenotype markers such as interleukin-4 receptor (IL-4R) and CD206 (Fig. 4F). In mouse infarcted myocardium, more CD206(+) cells were observed in the MI+5AZ group than in the MI+PBS group (Fig. 4G).

We then examined the expression of IRF1 in the infiltrated macrophages in the infarcted myocardium by double fluorescence immunostaining. Owing to technical difficulty with antibody reactivity, we performed immunostaining for CD68 and IRF1 in a rat MI model. CD68(+) cells were identified as macrophages, and IRF1 protein was stained by green fluorescence. Compared with the MI+PBS group, the MI+5AZ group showed more abundant CD68(+) IRF1(+) cells (Fig. 4H, white arrows). These double immunostaining results showed that more IRF1-expressing macrophages were observed in the MI+5AZ group than in the MI+PBS group.

### 5AZ potentiates sumoylation of IRF1 to exert resistance to degradation

**Figure 5:**
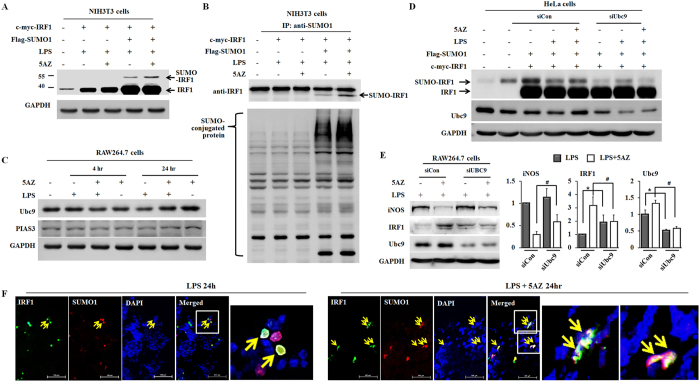
5AZ potentiates sumoylation of IRF1. (**A**) NIH3T3 cells were transfected with flag-SUMO-1 and c-myc-IRF1 for 24 hours and were then treated with LPS with or without 5AZ for a further 24 hours. Cell extracts were blotted with anti-IRF1 antibody. Longer exposure revealed sumoylated IRF1 bands. (**B**) NIH3T3 cells were transfected with flag-SUMO-1 and c-myc-IRF1 for 24 hours and were then treated with LPS with or without 5AZ for a further 24 hours. Cell extracts were precipitated with anti-SUMO-1 antibody and blotted with anti-IRF1 or anti-flag antibody. (**C**) Ubc9 protein level was reduced by LPS treatment, while upregulated in the presence of 5AZ. On the other hand, PIAS3 did not show a significant change. (**D**) HeLa cells were transfected with flag-SUMO-1, c-myc-IRF1, and control siRNA or UBC9 siRNA for 24 hours and were then treated with LPS with or without 5AZ for a further 24 hours. Cell extracts were blotted with anti-IRF1 antibody. Longer exposure revealed sumoylated IRF1 bands. (**E**) RAW264.7 cells were transfected with siRNA control (siCon) or siRNA Ubc9 (siUbc9) for 24 hours, and further treated with LPS with or without 5AZ. Immunoblots were performed and quantitated. *Significantly different from siCon-transfected cells treated with LPS, p < 0.05. ^#^Significantly different from siCon-transfected cells treated with LPS and 5AZ, p < 0.05. (**F**) c-myc-IRF1 and flag-SUMO-1 were transfected into 293T cells. Cells were treated with LPS or LPS + 5AZ for 24 hours, and localization of IRF1 or flag was visualized by fluorescence immunostaining.

Prolonged accumulation of IRF1 by 5AZ should be addressed in regard to the inhibition of iNOS induction. Because several researchers have reported that sumoylated IRF1 represses the transcriptional activity of IRF1^14^,^15^,^16^,^17^, we examined whether IRF1 sumoylation was modulated by 5AZ treatment. We transfected c-myc-IRF1 and flag-SUMO-1 to NIH3T3 cells for 24 hours and then treated cells with LPS with or without 5AZ for another 24 hours. Sumoylated IRF1 was observed by longer exposure in immunoblotting and was increased by 5AZ treatment (Fig. 5A).

To further confirm the IRF1 sumoylation, immunoprecipitation was performed after transfection of c-myc-IRF1 and flag-SUMO-1 followed by LPS stimulation with or without 5AZ. Cell extract was immunoprecipitated with anti-SUMO-1 antibody and immunoblotted with anti-IRF1 antibody. The level of sumoylated IRF1 was increased by 5AZ (Fig. 5B).

Ubc9 and PIAS3 are SUMO-1 conjugating enzymes essential for sumoylation of IRF1. LPS treatment lowered the levels of Ubc9, while was strikingly upregulated in the presence of 5AZ. On the other hand, PIAS3 was not changed significantly (Fig. 5C).

To confirm the relationship of Ubc9 and IRF1 sumoylation by 5AZ, the effect of Ubc9 silencing was studied by using siRNA. Immunoblot was performed as Fig. 5A in the absence or presence of Ubc9 siRNA transfection to HeLa cells. The protein levels of Ubc9 were reduced and 5AZ-induced IRF1 sumoylation was not increased in the presence of siUbc9 (Fig. 5D).

To investigate the impact of Ubc9-mediated modification of IRF1 on iNOS inhibition in RAW264.7 cells, we measured the effect of 5AZ on protein levels of iNOS and IRF1 after Ubc9 silencing (Fig. 5E). Immunoblot analyses confirmed that the inhibitory effect of 5AZ on iNOS was blunted by Ubc9 knockdown in RAW264.7 cells. 5AZ reduced iNOS to 29.4% in siCon-transfected cells, whereas reduced to 51.56% in siUbc9-transfected cells (*p* < 0.05). As anticipated, 5AZ-induced IRF1 upregulation was reduced by Ubc9 knockdown. From these results, the inhibition of LPS-induced iNOS by 5AZ was suggested to be mediated by Ubc9. On the other hand, IRF1 was upregulated by Ubc9 knockdown in LPS-stimulated RAW264.7 cells. We observed an unexpected upregulation of IRF1 on Western blots of siUbc9-transfected cells compared with siCon-transfected RAW264.7 cells (Fig. 5E). A recent report also showed that interferon-γ-stimulated gene products such as CXCL10, IRF1, and TAP1 were enhanced by Ubc9 depeletion^18^.

Next, double fluorescence immunostaining was performed with antibodies against IRF1 and flag in c-myc-IRF1 and flag-SUMO-1 transfected 293T cells. IRF1(+) flag(+) cells were more abundant in the 5AZ-treated cells than in the PBS-treated cells (Fig. 5F). In transfected cells, more co-localizations of IRF1 and flag-SUMO-1 were observed in 5AZ-treated cells than in PBS-treated cells.

## Discussion

The number of resident macrophages significantly increases during cardiac damage^1^,^3^. In the aftermath of cardiac injury, a large number of M1 macrophages are rapidly recruited to contribute to cardiac remodeling in the inflammatory phase. After this period, the infiltrated macrophages display a predominant M2 phenotype and promote cardiac repair by mediating profibrotic responses in the reparative phase^4^,^19^. As noted by these studies, the possibility of translational applications of these diverse phenotypes and dynamic adaptations have put macrophages in the spotlight.

This study was motivated by our previous findings that 5AZ modulates macrophage phenotypes by blocking iNOS induction and preventing cardiac contractile decompensation and decreased cardiac fibrosis after MI^5^. 5AZ, a DNA methylation inhibitor, is currently used for treating myelodysplastic syndrome and high-risk leukemia^20^ and is being clinically studied for applications for various tumors (www.clinicaltrials.gov).

IRFs were initially identified to be involved in immune responses, tumor suppression, and apoptosis in various cell types. Recent intensive studies have demonstrated the diverse roles of IRF family members in cardiac-specific IRF transgenic mice. IRF3, IRF7, IRF8, and IRF9 show a protective effect against cardiac hypertrophy^21^,^22^,^23^,^24^, whereas IRF1 and IRF4 function as adverse factors in cardiac hypertrophy, fibrosis, and cardiac dysfunction^25^,^26^. Those studies explained the functional role of IRFs in cardiomyocytes in response to pressure overload. In the present study, we found that 5AZ altered the response of IRF1 in activated macrophages, which exerted a protective effect on cardiac dysfunction after MI.

Although IRF1 protein is usually degraded shortly after translational induction, we observed the accumulation of IRF1 in the presence of 5AZ. IRF1 is known to be phosphorylated by casein kinase II and IκB kinase ε, and sumoylated by PIAS3, and Ubc9 to exert diverse cellular functions^15^,^27^,^28^. Posttranslational modification by SUMO is implicated in the dynamic control of cellular processes and rapid reaction to environmental changes without *de novo* protein synthesis. The major finding of the present study was that 5AZ inhibited iNOS and simultaneously made IRF1, a transcription factor for iNOS, resistant to proteasomal degradation through sumoylation in macrophages. Consequently, sumoylated IRF1 might repress the transcriptional activity of IRF1^5^,^10^, and we suggest that 5AZ potentiated IRF1 sumoylation.

The iNOS promoter is activated by the binding of several transcriptional activators containing NF-κB, IRF1, and C/EBPβ to their respective cognitive sites. Our results did not show the involvement of p65, p50, or p52 in the action of 5AZ (Fig. S1C,D). The 5’-flanking region of the iNOS gene contains two clusters of *cis*-acting regulatory elements that are essential for iNOS transcription. The proximal cluster of elements is required for LPS-induced activation and the distal cluster is essential for IFN-γ-induced activation^29^. There are IRF binding sites G(A)AAA(G/C)(T/C)-GAAA(G/C)(T/C) on the 5’-flanking region of the iNOS promoter^17^, and we thus decided to focus on IRF1 as a target of 5AZ in macrophages.

IRF1 protein is short-lived (*t*_0.5_ ~30 min) and has been shown to be multi-ubiquitinated and then rapidly degraded by the proteasome^30^. The peak induction of IRF1 protein occurred at 4 hours of LPS stimulation, although iNOS induction was still sustained slightly above basal levels after 4 hours. The induced IRF1 protein then decreased over time. In the presence of 5AZ, IRF1 was induced in response to LPS without any interference, and this increase was significantly sustained up to 24 hours.

The additional fluorescence staining showed that increased IRF1 accumulated in the nuclei 4 hours after LPS stimulation regardless of 5AZ treatment. At 24 hours, upregulated IRF1 protein was significantly sustained in the nuclei in the presence of 5AZ, but almost disappeared in the absence of 5AZ (Fig. 2A).

In the animal study, we confirmed the functional and histological recovery after MI by 5AZ treatment. 5AZ treatment apparently contributed to not only the reduction of the macrophages (Fig. 4C) but a shift toward anti-inflammatory phenotype in the infarcted myocardium^5^. In terms of the circulating monocytes, the number of monocytes in the blood showed a tendency to increase after MI as previously reported^4^. The effect of 5AZ on monocytes in the blood was evaluated by quantification. These results showed that 5AZ treatment did not exert a significant effect on the number of circulating monocytes (Fig. 4D,E)

To confirm the upregulation of IRF1 *in vivo*, we examined IRF1 expression in infiltrated macrophages in infarcted myocardium. IRF1-expressing macrophages, identified by CD68, were more frequently present in 5AZ-treated infarcted myocardium (Fig. 4H).

On the basis of these data, we hypothesized that the prolonged maintenance of IRF1 protein implied the involvement of an antagonist of IRF1. To find the functional repressor of IRF1, we first checked the levels of IRF2 and then the sumoylation of IRF1. First, IRF2 negatively regulates the gene expression induced by IRF1 through competitive binding to the target gene promoters^14^. In this study, IRF2 was irrelevant to both LPS stimulation and 5AZ treatment (Fig. 1E).

Second, sumoylated IRF1 is reported to be resistant to ubiquitination, which thus interferes with IRF1 activity^5^,^15^,^31^.

Sumoylation is a covalent modification of IRF1 protein by PIAS3^14^, and Ubc9^10^. Sumoylation mediates functional changes in IRF1 by altering protein-protein interaction and subcellular localization or by regulating the ubiquitination of target proteins. In the present study, forced expression of IRF1 and SUMO1 resulted in an increased level of sumoylated IRF1 in cells in the presence of 5AZ. Although 5AZ-induced IRF1 sumoylation shows to be dependent on Ubc9 for iNOS inhibition, further studies are required to identify other sumoylated proteins and define their roles in the progress of inflammation.

**Figure 6:**
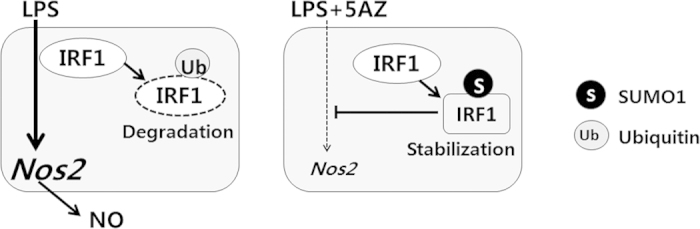
Proposed model of IRF1 sumoylation in LPS-stimulated macrophages. LPS induces iNOS via induction of IRF1, which is subsequently degraded via ubiquitination in activated macrophages. In the presence of 5AZ, LPS-induced IRF1 is sumoylated to be resistant to degradation. Sumoylated IRF1 might be involved in the inhibitory effect of 5AZ on iNOS induction.

The suggested mode of action of 5AZ is illustrated in Fig. 6. 5AZ is proposed to inhibit iNOS induction through Ubc9-mediated IRF1 sumoylation in activated macrophages.

The present study has shown that increased sumoylation of IRF1 alters its role and leads to the loss of iNOS induction, which allows macrophages to shift to the anti-inflammatory phenotype. This finding illustrates a causative mechanism of 5AZ action in macrophages. Future studies will be critical to fully characterize the role of IRF1 in macrophage transformation as well as the specific role of 5AZ in cardiac pathology.

Importantly, given its clinical availability, we suggest 5AZ as a compound for resolving post-MI inflammation. Translation of our concept into clinical studies might open a new window for safe and successful cardiac repair.

## Materials and Methods

### Reagents

Antibodies against iNOS, CD206, Ubc9, PIAS3, and F4/80 were purchased from Abcam (Abcam, Cambridge, UK); IRF1, IRF2, IκBα, phosphorylated p65, phosphorylated signal transducers and activators of transcription 1 (STAT1), and SUMO-1 were from Cell Signaling Technology (Danvers, MA, USA); p65, p50, p52, PCNA, Stat1 p84/p91, and GAPDH were from Santa Cruz Biotechnology (Santa Cruz Biotechnology, Inc., Santa Cruz, CA, USA); Flag, and c-Myc were from Sigma (Sigma-Aldrich Corp., St. Louis, MO, USA); CD68 was from BMA Biomedicals (BMA Biomedicals, Augst, Switzerland); CD45-PE and CD14-PC5 were from Beckman Coulter (Brea, CA, USA). Anti-mouse or anti-rabbit IgG peroxidase-conjugated secondary antibodies, Alexa Fluor 488-conjugated anti-mouse IgG, and Alexa 568-conjugated anti-rabbit IgG secondary antibodies were purchased from Invitrogen (Invitrogen Life Technologies, Carlsbad, CA, USA). Protein A/G was from Wanta Cruz Biotechnology. 5-Azacytidine (5AZ), lipopolysaccharide (LPS), methylthiazolyldiphenyl-tetrazolium bromide (MTT), and MG132 were purchased from Sigma (Sigma-Aldrich Corp.).

### Cell culture and stimulation

The RAW264.7 murine monocyte/macrophage cell line, NIH3T3 cells, HeLa cells, and 293T cells were purchased from Korean Cell Line Bank (Seoul, Korea) and were cultured in Dulbecco’s Modified Eagle’s Medium (Invitrogen Life Technologies) supplemented with 10% heat-inactivated fetal bovine serum. RAW264.7 cells were treated with LPS (100 ng/mL) with or without 5AZ (10 μM). Cells were treated with MG132 (10 μM) to block the proteasomal degradation. Cell viability was measured by MTT assay. Briefly, cells were incubated with MTT solution (0.5 mg/mL) for 4 hours, media was removed and further incubated with DMSO to measure the absorbance at 550 nm.

### Measurement of nitric oxide

RAW264.7 cells were seeded in 24-well culture plates and stimulated with LPS with or without 5AZ to measure the amount of NO production. Culture supernatants were collected by centrifugation (10000 × *g* for 5 minutes) and were assayed for NO production by use of Griess reagent according to the manufacturer's instructions (Promega, Madison, WI, USA).

### RNA isolation and RT-PCR

To compare mRNA expression levels, cells were harvested and homogenized in Trizol solution (Invitrogen Life Technologies) according to the manufacturer’s instructions. cDNA was synthesized to perform RT-PCR. GAPDH was used as a loading control. The sequences of primer pairs are described in Supplementary Table S1.

### Western blot and immunoprecipitation

Cells were washed with ice-cold phosphate-buffered PBS (PBS), resuspended in lysis buffer (20 mM Tris-HCl, pH7.4, 0.1 mM EDTA, 150 mM NaCl, 1 mM phenylmethylsulfonyl fluoride, 1 mg/mL leupeptin, 1 mM Na_3_VO_4_), and sonicated briefly. After centrifugation at 10000 × *g* for 10 minutes, the supernatant was prepared as a protein extract. Equal concentrations of proteins were fractionated by electrophoresis on 8% or 10% acrylamide gels and were transferred onto a polyvinylidene fluoride membrane (PVDF, Thermo Scientific, Rockford, IL, USA) followed by blotting with antibodies. Protein levels were determined by using Western Breeze reagents (Santa Cruz Biotechnology) and an Image Reader (LAS-3000 Imaging System, Fuji Photo Film, Japan). For immunoprecipitation, cells were lysed and centrifuged at 12,000g for 15 minutes at 4 °C. The supernatants were incubated with the appropriated antibodies for 2 hours at 4 °C and protein G/A-conjugated beads were added in for additional 1 hour. Precipitates were collected and washed four times with lysis buffer, and then proteins were eluted with SDS-PAGE sample buffer.

### Plasmids and siRNA

iNOS-promoter-luciferease was kindly gifted by Professor Myung-Jun Kim (College of Medicine, Catholic University, Seoul, Korea). IRF1 clone was provided by Korea Human Gene Bank, Medical Genomics Research Center, KRIBB (Daejoen, Korea) and was transferred to the pcDNA3.1-c-myc expression vector. Flag-SUMO-1 was kindly provided by Jae Bum Kim (Seoul National University, Seoul, Korea). Plasmids were transfected by using Lipofectamine 2000 (Invitrogen Life Technologies). Small interfering RNAs targeting Ubc9 purchased from Bioneer (Bioneer, Daejeon, Korea). siRNA duplexes were transfected into HeLa cells or RAW264.7 cells using Lipofectamine RNAiMAX (Invitrogen Life Technologies).

### Promoter study

RAW264.7 cells were transfected with iNOS-promoter-luciferase in triplicate by using Effectene (Qiagen, Venlo, Netherlands) according to the manufacturer’s protocol. Twenty-four hours after transfection, cells were washed with PBS, and treated with LPS with or without 5AZ. Cells were harvested and luciferase activity was assayed by using the Dual Luciferase Reporter assay system with normalization to Renilla luciferase activity (Promega, Madison, WI).

### Immunofluorescent staining

To analyze the cellular location of IRF1 in RAW264.7 cells, cells were fixed with 4% paraformaldehyde for 15 minutes and were washed 3 times with PBS for 5 minutes each. After permeabilization by 0.1% Triton X-100 for 10 min and blocking with 5% normal goat serum in the room temperature for 1 hour, excess serum was removed and the cells were incubated overnight at 4 °C with IRF1 antibody, followed by incubation with Alexa-Fluor 594 secondary antibody for 1 hour. Coverslips were mounted in ProLong Gold Antifade Reagent with DAPI (Invitrogen Life Technologies) to identify nuclei. Cells were then photographed with a fluorescent microscope (Olympus, Tokyo, Japan).

### Mouse model of myocardial infarction and quantification of circulating monocytes

To investigate the role of 5AZ, MI was induced in mice by permanent ligation of the left anterior descending coronary artery. The study was reviewed and approved by the Chonnam National University Institutional Animal Care and Use Committee (CNU IACUC-H-2010-12). Eight-week-old male BALB/c mice were purchased from Jung Ang Animals (Seoul, Korea). Animals were anesthetized with an intramuscular injection of ketamine (50 mg/kg) and xylazine (5 mg/kg), intubated, and mechanically ventilated. The proximal left anterior descending coronary artery was ligated. Finally, the heart was repositioned in the chest, and the chest was closed. The animals remained in a supervised setting until becoming fully conscious. After 1 day of MI, the mice were administered PBS (n = 6) or 5AZ (5 mg/kg of body weight in PBS, n = 6) every other day via intraperitoneal injection for 2 weeks. For quantification of circulating monocytes, animals were sacrificed and whole blood was collected in EDTA-tube. Automated WBC differential was processed on XE-2100 (Sysmex, Kobe, Japan), and the number of CD45(+)CD14(+) monocytes was calculated on flow cytometry (Beckman Couter Inc., USA) by an experienced hematopathologist. Monocytes were discovered by microscopic examination of peripheral blood stained with Wright's stain. All procedures were performed in accordance with approved guidelines from the Institutional Animal Care and Use Committee, Chonnam National University.

### Rat model of myocardial infarction

The study was reviewed and approved by the Chonnam National University Institutional Animal Care and Use Committee (CNU IACUC-H-2010-12). Male Sprague-Dawley rats (weighing 200–230 g) were purchased from Jung Ang Animals (Seoul, Korea). For MI induction, rats were anesthetized with an intramuscular injection of ketamine (50 mg/kg) and xylazine (10 mg/kg), intubated, and mechanically ventilated. The proximal left anterior descending coronary artery was ligated. Finally, the heart was repositioned in the chest, and the chest was closed. The animals remained in a supervised setting until becoming fully conscious. After 1 day of MI, rats were administered with PBS or 5AZ (2.5 mg/kg of body weight in PBS) every other day via intraperitoneal injection for 2 weeks. All procedures were performed in accordance with approved guidelines from the Institutional Animal Care and Use Committee, Chonnam National University.

### Left ventricular function measurement

Left ventricular function was assessed by echocardiography. After 2 weeks, the animals were anesthetized and intubated and echocardiography was performed to measure left ventricular function. Echocardiographic studies were performed with a 15-MHz linear array transducer system (iE33 system, Philips Medical Systems) by an expert who was not aware of the experimental conditions to exclude bias. Two-dimensional guided M-mode of the LV was obtained from the parasternal view.

### Immunohistochemical staining

At the end of the experiment, the animals were sacrificed, and the hearts were rapidly removed, fixed in formalin, and embedded in paraffin for histological studies. For immunohistochemical analysis, slides were treated with 3% hydrogen peroxide in PBS for 10 minutes at room temperature to block endogenous peroxidase activity. After nonspecific binding was blocked with 5% normal goat serum (Sigma-Aldrich Corp.), the slides were incubated with primary antibodies for 18 hours at 4 °C. Sections were washed with PBS three times, and then incubated for 1 hour with Alexa-Fluor 488 or 594 secondary antibodies. After washing, the slides were coverslipped with mounting medium (VectaMount mounting medium, Vector Labs Inc., Burlingame, CA, USA). Images were obtained and digitized on a computer by using an Olympus CX31 microscope (Olympus) equipped with an Infinity 1 camera (Lumenera Scientific, Ottawa, Canada). Immunofluorescence was detected by using a Carl-Zeiss confocal microscope. Images were obtained by using Zeiss LSM version 3.2 SP2 software. Cardiac fibrosis was measured by Masson’s trichrome staining, and fibrotic areas were measured by visualizing blue-stained fibrotic deposits by using the NIS-Elements Advanced Research program (Nikon, Japan). The percentage of ventricular fibrosis was calculated as the blue-stained area divided by total ventricular area.

In mouse myocardium, infiltrated macrophages were hardly detected by double-fluorescence immunohistochemical staining by using CD68, F4/80, or Mac-2 antibodies. On the other hand, CD68 worked well to detect macrophages in rat myocardium as previously reported^5^. Owing to this problem, we utilized rat infarcted myocardium for identifying IRF1-expressing macrophages.

### Statistical analysis

The data are presented as means ± SD. Differences were analyzed by an unpaired Student’s t-test. A P value of less than 0.05 was considered significant.

## Additional Information

**How to cite this article**: Jeong, H.- *et al.* 5-Azacytidine modulates interferon regulatory factor 1 in macrophages to exert a cardioprotective effect. *Sci. Rep.* **5**, 15768; doi: 10.1038/srep15768 (2015).

## Supplementary Material

Supplementary Information

## Figures and Tables

**Table 1:** Echocardiographic evaluation at two weeks after MI.

	Non MI + PBS	Non MI + 5AZ	MI + PBS	MI + 5AZ
IVSd (mm)	0.575 ± 0.046	0.625 ± 0.045	0.633 ± 0.052	0.650 ± 0.054
IVSs (mm)	0.600 ± 0.053	0.642 ± 0.116	0.617 ± 0.075	0.638 ± 0.074
LVIDd (mm)	3.575 ± 0.128	3.529 ± 0.154	3.683 ± 0.319	3.800 ± 0.151
LVIDs (mm)	2.188 ± 0.136	2.167 ± 0.115	2.850 ± 0.259	2.700 ± 0.160
LVPWd (mm)	0.525 ± 0.071	0.558 ± 0.067	0.617 ± 0.041	0.638 ± 0.092
LVPWs (mm)	0.780 ± 0.120	0.730 ± 0.100	0.700 ± 0.110	0.880 ± 0.150*
EF (%)	75.213 ± 2.368	75.851 ± 1.111	53.250 ± 2.551	62.503 ± 7.200*
FS (%)	38.300 ± 2.097	38.790 ± 0.978	23.220 ± 1.410	28.710 ± 4.073*

*p < 0.05 vs. MI+PBS group.

IVSd, intraventricular septal width in diastole; IVSs, intraventricular septal width in systole; LVIDd, left ventricular internal dimension in diastole; LVIDs, left ventricular internal dimension in systole; LVPWd, left ventricular posterior wall thickness in diastole; LVPWs, left ventricular posterior wall thickness in systole; EF, ejection fraction; FS, fractional shortening.

## References

[b1] Nahrendorf M., Pittet M. J. & Swirski F. K. Monocytes: protagonists of infarct inflammation and repair after myocardial infarction. Circulation 121, 2437–2445, 10.1161/CIRCULATIONAHA.109.916346 (2010). 20530020 PMC2892474

[b2] Gordon S. & Taylor P. R. Monocyte and macrophage heterogeneity. Nature reviews. Immunology 5, 953–964, 10.1038/nri1733 (2005). 16322748

[b3] Troidl C. *et al.* Classically and alternatively activated macrophages contribute to tissue remodelling after myocardial infarction. Journal of cellular and molecular medicine 13, 3485–3496, 10.1111/j.1582-4934.2009.00707.x (2009). 19228260 PMC4516503

[b4] Hilgendorf I. *et al.* Ly-6Chigh monocytes depend on Nr4a1 to balance both inflammatory and reparative phases in the infarcted myocardium. Circulation research 114, 1611–1622, 10.1161/CIRCRESAHA.114.303204 (2014). 24625784 PMC4017349

[b5] Kim Y. S. *et al.* Protective role of 5-azacytidine on myocardial infarction is associated with modulation of macrophage phenotype and inhibition of fibrosis. Journal of cellular and molecular medicine 18, 1018–1027, 10.1111/jcmm.12248 (2014). 24571348 PMC4508142

[b6] Kamijo R. *et al.* Requirement for transcription factor IRF-1 in NO synthase induction in macrophages. Science 263, 1612–1615 (1994). 7510419 10.1126/science.7510419

[b7] Spink J. & Evans T. Binding of the transcription factor interferon regulatory factor-1 to the inducible nitric-oxide synthase promoter. The Journal of biological chemistry 272, 24417–24425 (1997). 9305901 10.1074/jbc.272.39.24417

[b8] Honda K. & Taniguchi T. IRFs: master regulators of signalling by Toll-like receptors and cytosolic pattern-recognition receptors. Nature reviews. Immunology 6, 644–658, 10.1038/nri1900 (2006). 16932750

[b9] Tamura T., Yanai H., Savitsky D. & Taniguchi T. The IRF family transcription factors in immunity and oncogenesis. Annual review of immunology 26, 535–584, 10.1146/annurev.immunol.26.021607.090400 (2008). 18303999

[b10] Battistini A. Interferon regulatory factors in hematopoietic cell differentiation and immune regulation. Journal of interferon & cytokine research: the official journal of the International Society for Interferon and Cytokine Research 29, 765–780, 10.1089/jir.2009.0030 (2009). 19929577

[b11] Kirchhoff S., Schaper F. & Hauser H. Interferon regulatory factor 1 (IRF-1) mediates cell growth inhibition by transactivation of downstream target genes. Nucleic acids research 21, 2881–2889 (1993). 8332497 10.1093/nar/21.12.2881 PMC309674

[b12] Tamura T. *et al.* An IRF-1-dependent pathway of DNA damage-induced apoptosis in mitogen-activated T lymphocytes. Nature 376, 596–599, 10.1038/376596a0 (1995). 7637809

[b13] Kroger A., Koster M., Schroeder K., Hauser H. & Mueller P. P. Activities of IRF-1. Journal of interferon & cytokine research : the official journal of the International Society for Interferon and Cytokine Research 22, 5–14, 10.1089/107999002753452610 (2002). 11846971

[b14] Harada H. *et al.* Structurally similar but functionally distinct factors, IRF-1 and IRF-2, bind to the same regulatory elements of IFN and IFN-inducible genes. Cell 58, 729–739 (1989). 2475256 10.1016/0092-8674(89)90107-4

[b15] Nakagawa K. & Yokosawa H. PIAS3 induces SUMO-1 modification and transcriptional repression of IRF-1. FEBS letters 530, 204–208 (2002). 12387893 10.1016/s0014-5793(02)03486-5

[b16] Kim E. J., Park J. S. & Um S. J. Ubc9-mediated sumoylation leads to transcriptional repression of IRF-1. Biochemical and biophysical research communications 377, 952–956, 10.1016/j.bbrc.2008.10.092 (2008). 18955028

[b17] Park S. M. *et al.* SUMOylated IRF-1 shows oncogenic potential by mimicking IRF-2. Biochemical and biophysical research communications 391, 926–930, 10.1016/j.bbrc.2009.11.166 (2010). 19962964

[b18] Maarifi G. *et al.* Small Ubiquitin-like Modifier Alters IFN Response. J Immunol , 10.4049/jimmunol.1500035 (2015). 26223657

[b19] Heidt T. *et al.* Differential contribution of monocytes to heart macrophages in steady-state and after myocardial infarction. Circulation research 115, 284–295, 10.1161/CIRCRESAHA.115.303567 (2014). 24786973 PMC4082439

[b20] Kaminskas E. *et al.* Approval summary: azacitidine for treatment of myelodysplastic syndrome subtypes. Clinical cancer research: an official journal of the American Association for Cancer Research 11, 3604–3608, 10.1158/1078-0432.CCR-04-2135 (2005). 15897554

[b21] Lu J. *et al.* Interferon regulatory factor 3 is a negative regulator of pathological cardiac hypertrophy. Basic research in cardiology 108, 326, 10.1007/s00395-012-0326-9 (2013). 23307144

[b22] Jiang D. S. *et al.* Interferon regulatory factor 9 protects against cardiac hypertrophy by targeting myocardin. Hypertension 63, 119–127, 10.1161/HYPERTENSIONAHA.113.02083 (2014). 24144649

[b23] Jiang D. S. *et al.* Interferon regulatory factor 7 functions as a novel negative regulator of pathological cardiac hypertrophy. Hypertension 63, 713–722, 10.1161/HYPERTENSIONAHA.113.02653 (2014). 24396025 PMC5349187

[b24] Jiang D. S. *et al.* IRF8 suppresses pathological cardiac remodelling by inhibiting calcineurin signalling. Nature communications 5, 3303, 10.1038/ncomms4303 (2014). PMC3929801 24526256

[b25] Jiang D. S. *et al.* Interferon regulatory factor 1 is required for cardiac remodeling in response to pressure overload. Hypertension 64, 77–86, 10.1161/HYPERTENSIONAHA.114.03229 (2014). 24732887

[b26] Jiang D. S. *et al.* Role of interferon regulatory factor 4 in the regulation of pathological cardiac hypertrophy. Hypertension 61, 1193–1202, 10.1161/HYPERTENSIONAHA.111.00614 (2013). 23589561 PMC3734933

[b27] Lin R. & Hiscott J. A role for casein kinase II phosphorylation in the regulation of IRF-1 transcriptional activity. Molecular and cellular biochemistry 191, 169–180 (1999). 10094406

[b28] Sgarbanti M. *et al.* IkappaB kinase epsilon targets interferon regulatory factor 1 in activated T lymphocytes. Molecular and cellular biology 34, 1054–1065, 10.1128/MCB.01161-13 (2014). 24396068 PMC3958032

[b29] Lowenstein C. J. *et al.* Macrophage nitric oxide synthase gene: two upstream regions mediate induction by interferon gamma and lipopolysaccharide. Proceedings of the National Academy of Sciences of the United States of America 90, 9730–9734 (1993). 7692452 10.1073/pnas.90.20.9730 PMC47644

[b30] Nakagawa K. & Yokosawa H. Degradation of transcription factor IRF-1 by the ubiquitin-proteasome pathway. The C-terminal region governs the protein stability. European journal of biochemistry / FEBS 267, 1680–1686 (2000). 10.1046/j.1432-1327.2000.01163.x 10712599

[b31] Park J. *et al.* Elevated level of SUMOylated IRF-1 in tumor cells interferes with IRF-1-mediated apoptosis. Proceedings of the National Academy of Sciences of the United States of America 104, 17028–17033, 10.1073/pnas.0609852104 (2007). 17942705 PMC2040422

