# Supplementary material for: 5-Azacytidine modulates interferon regulatory factor 1 in macrophages to exert a cardioprotective effect
Source: Sci Rep. 2015 Oct 29;5:15768. doi: 10.1038/srep15768 (PMC4625165; doi:10.1038/srep15768)
Supplement: Supplementary Information [file srep15768-s1.doc]

**5-Azacytidine modulates interferon regulatory factor 1 in macrophages to exert a cardioprotective effect**

Hye-yun Jeong^a,b^, Wan Seok Kang^a,b^, Moon Hwa Hong^a^, Hae Chang Jeong^c^, Myun-Geun Shin^d^, Myung Ho Jeong^c^, Yong Sook Kim^a,e^,*, Youngkeun Ahn^a,c^,*

^a^Research Laboratory of Cardiovascular Regeneration, Chonnam National University Hospital, Gwangju, Republic of Korea

^b^Department of Molecular Medicine, Graduate School, Chonnam National University, Gwangju, Republic of Korea

^c^Department of Cardiology, Chonnam National University Hospital, Gwangju, Republic of Korea

^d^Department of Laboratory Medicine, Chonnam National University Hwasun Hospital, Hwasun, Republic of Korea

^e^Biomedical Research Center, Chonnam National University Hospital, Gwangju, Republic of Korea

*Addresses for correspondence:

**Youngkeun Ahn**, MD, PhD

e-mail) cecilyk@hanmail.net

Tel) 82-62-220-4764, Fax) 82-62-224-4764

Department of Cardiology, Chonnam National University Hospital, 671 Jebong-ro, Dong-gu, Gwangju 501-757, Republic of Korea

**Yong Sook Kim**, PhD

e-mail) retissue@chonnam.ac.kr

Tel) 82-62-220-5820, Fax) 82-62-223-3105

Biomedical Research Institute of Chonnam National University Hospital, 671 Jebong-ro, Dong-gu, Gwangju 501-757, Republic of Korea

**
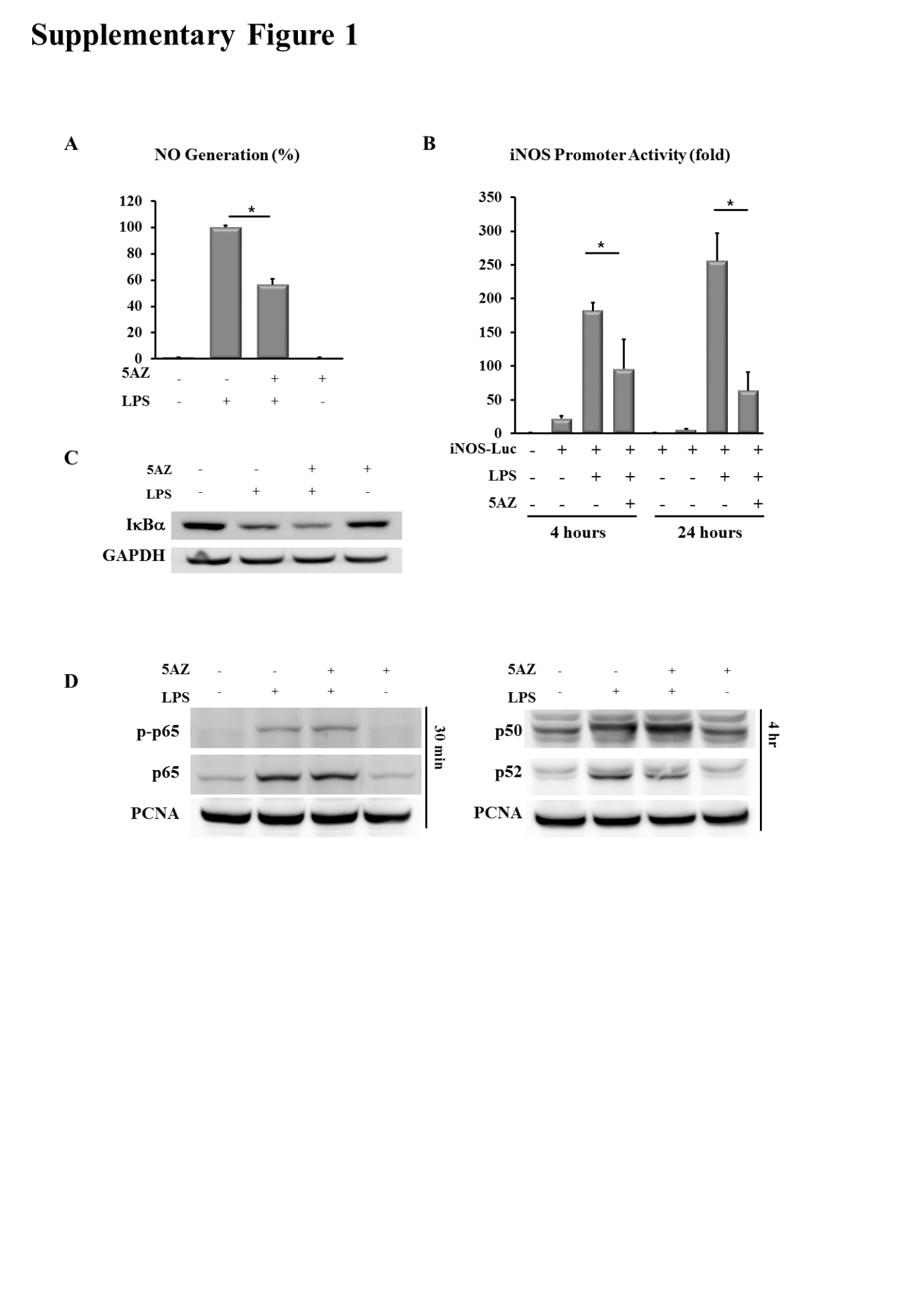
**

**Figure S1. 5AZ attenuates nitrite formation and iNOS promoter activity but has no effect on NF-κB.**

(A) RAW264.7 cells were treated with LPS in the absence or presence of 5AZ (10 μM) for 24 hours. Nitrite formation was measured, and the value from the cells stimulated by LPS alone for 24 hours was set to 100%. Results expressed as mean ± SD. * p < 0.05.

(B) Cells were transfected with iNOS-luciferase reporter plasmid. Twenty-four hours later, transfected cells were treated with LPS in the absence or presence of 5AZ for 4 hours or 24 hours, and luciferase activity was measured. The values are expressed as percentage of LPS-treated control. Data are shown as means ± SDs of triplicates and are representative of three independent experiments. Results expressed as mean ± SD. * p < 0.05.

(C) IκBα protein was reduced by LPS treatment for 30 minutes. 5AZ did not influence the LPS-induced IκBα degradation.

(D) Nuclear translocation of p65 and phosphorylated p65 in the nucleus fraction were induced by LPS treatment and were not changed by 5AZ treatment. LPS-induced nuclear translocation of p50 and p52 were not changed by 5AZ treatment.

**
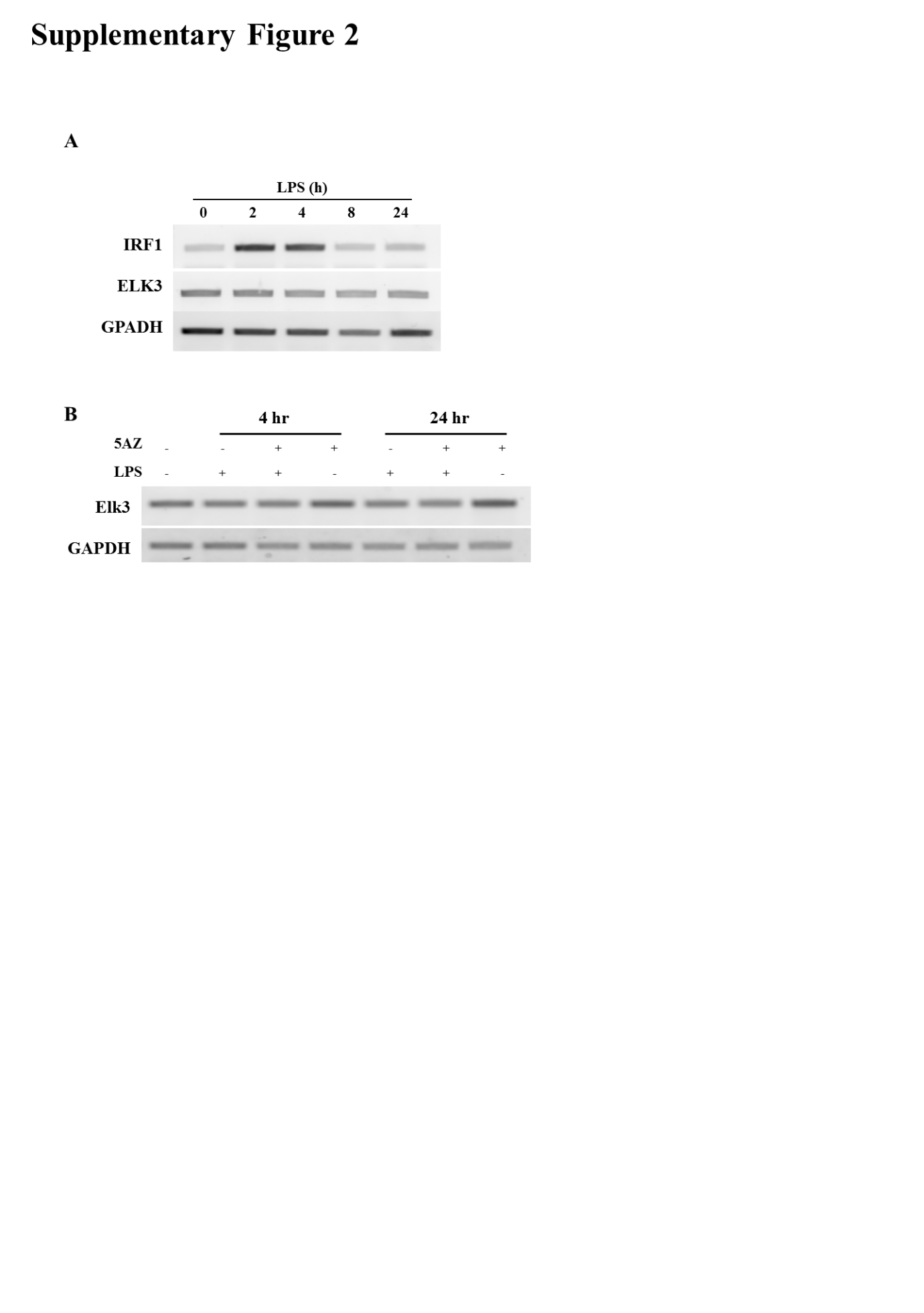
**

**Figure S2. Elk3, a negative regulator of iNOS, is not involved in the effect of 5AZ on iNOS induction.**

(A) ELK3 mRNA level was not changed, while IRF1 mRNA was induced during LPS stimulation in RAW264.7 cells.

(B) ELK3 mRNA was not changed in the presence of 5AZ in LPS-stimulated RAW264.7 cells.

**
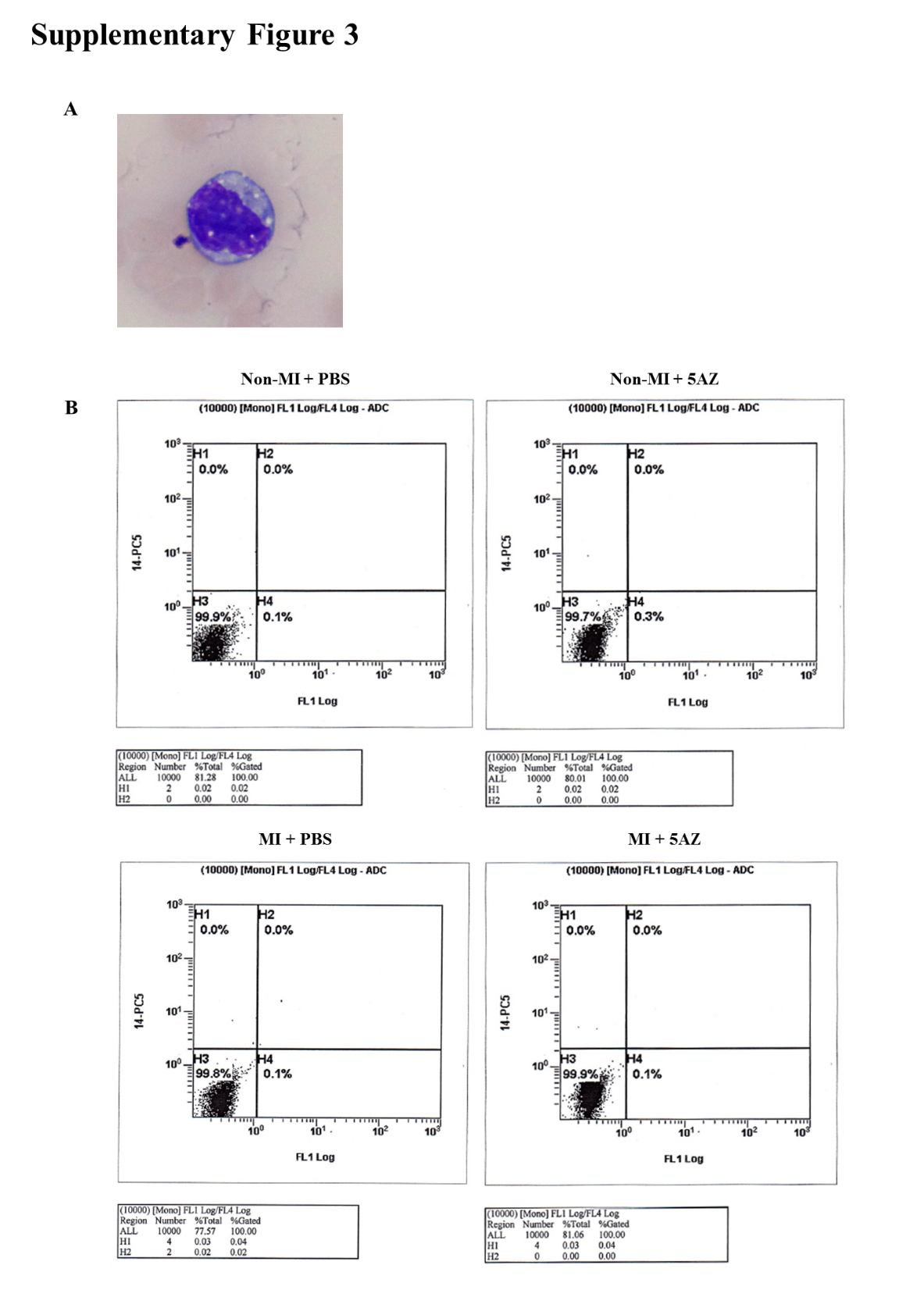
**

**Figure S3. Circulating monocytes are quantified by flow cytometric analysis.**

(A) A Wright-stained peripheral blood smear was evaluated.

(B) The relative proportion of monocytes is determined by flow cytometry and the representative cytograms are shown.

**Figure S4. The cropped immunoblots in the main figures are indicated with boxes.**


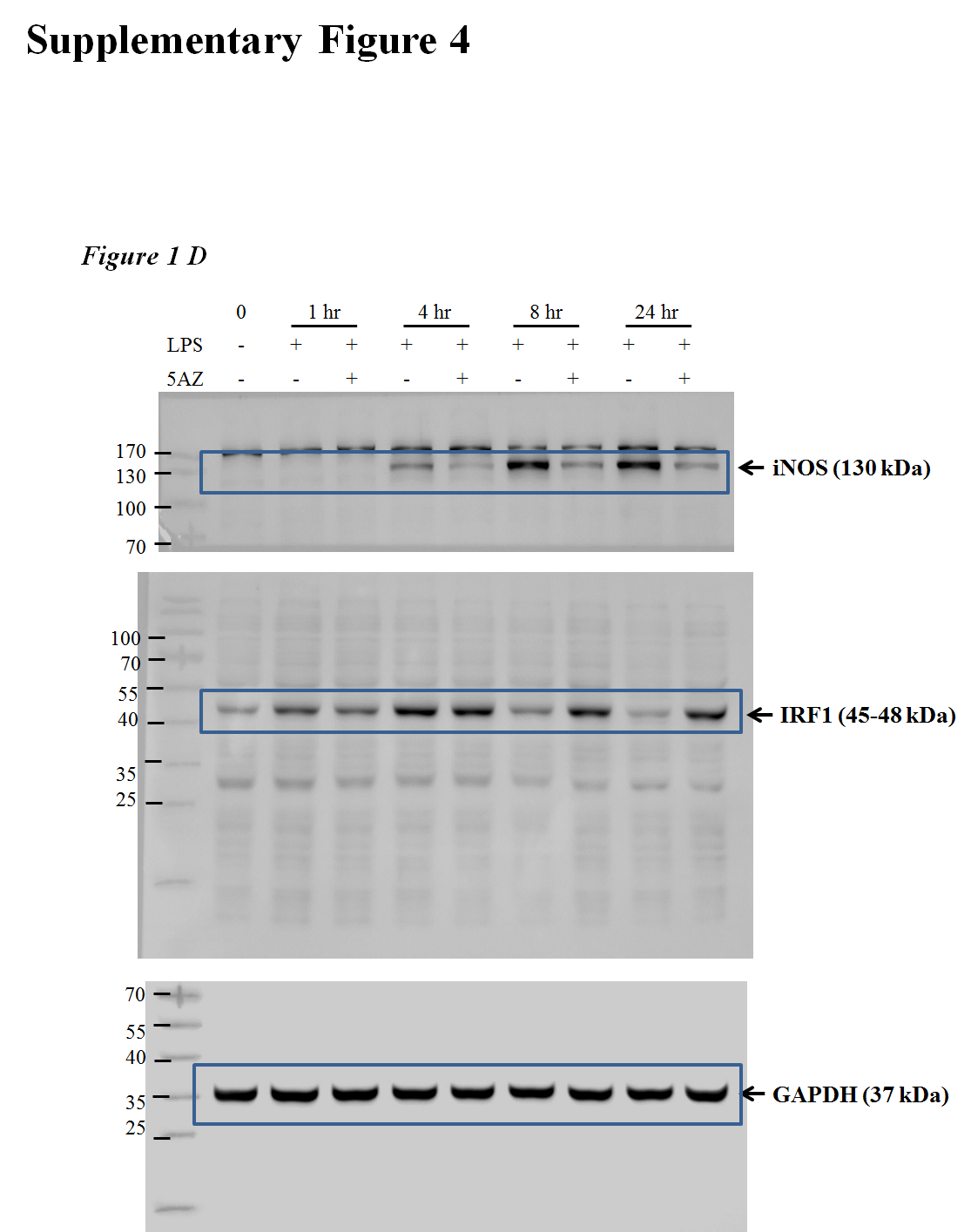
Original immunoblots were shown with figure numbers in the manuscript.

**
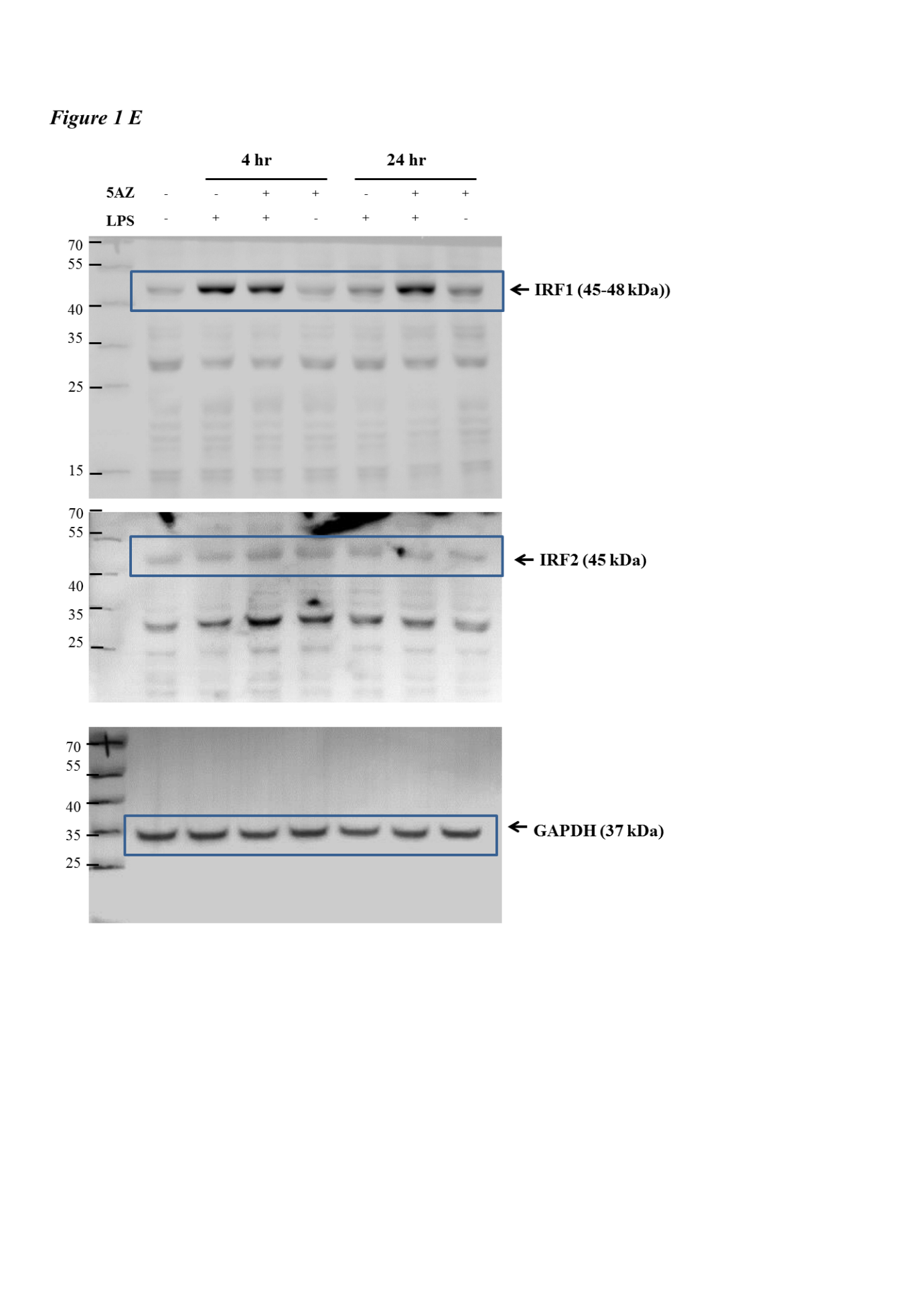
**

**
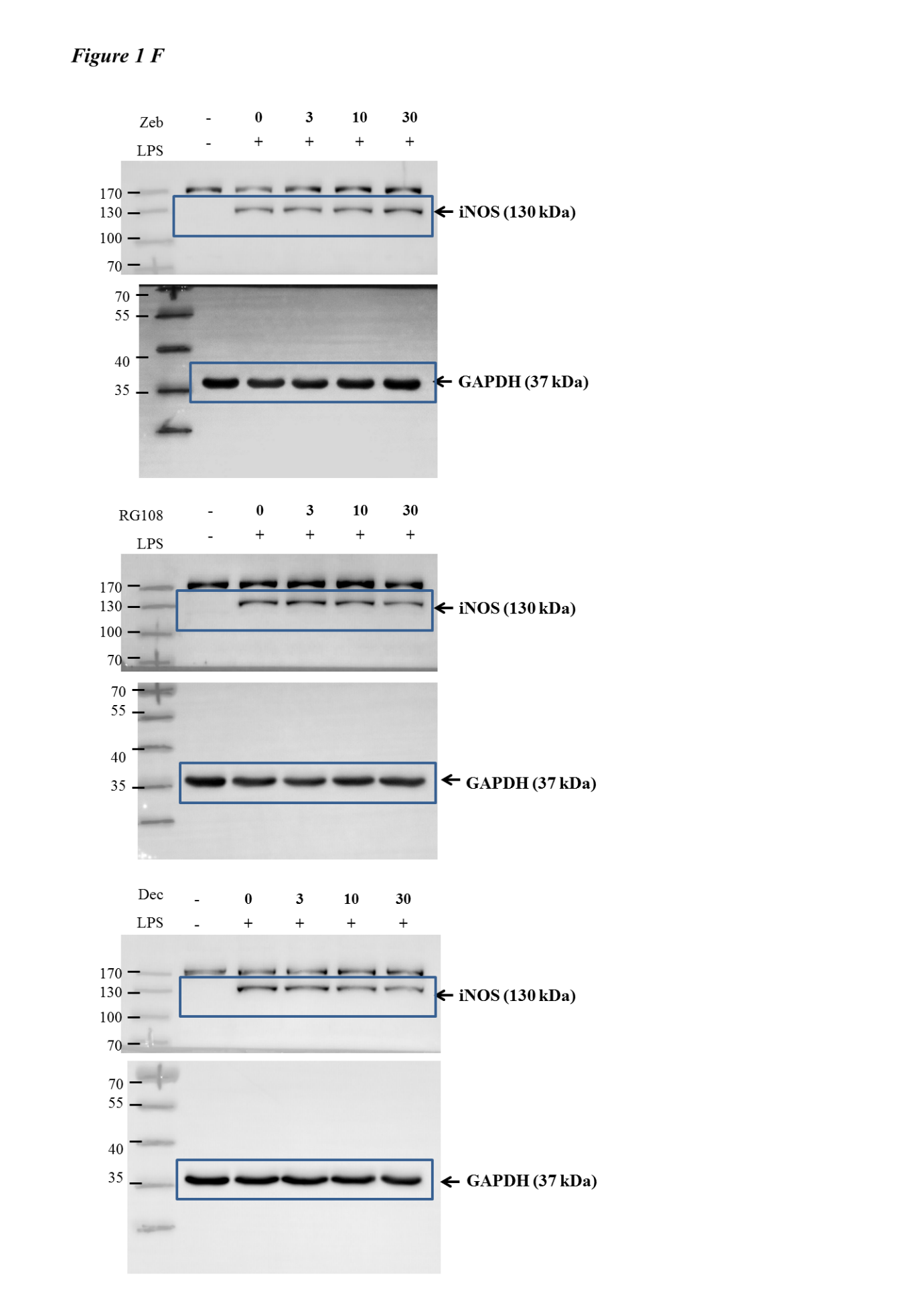
**

**
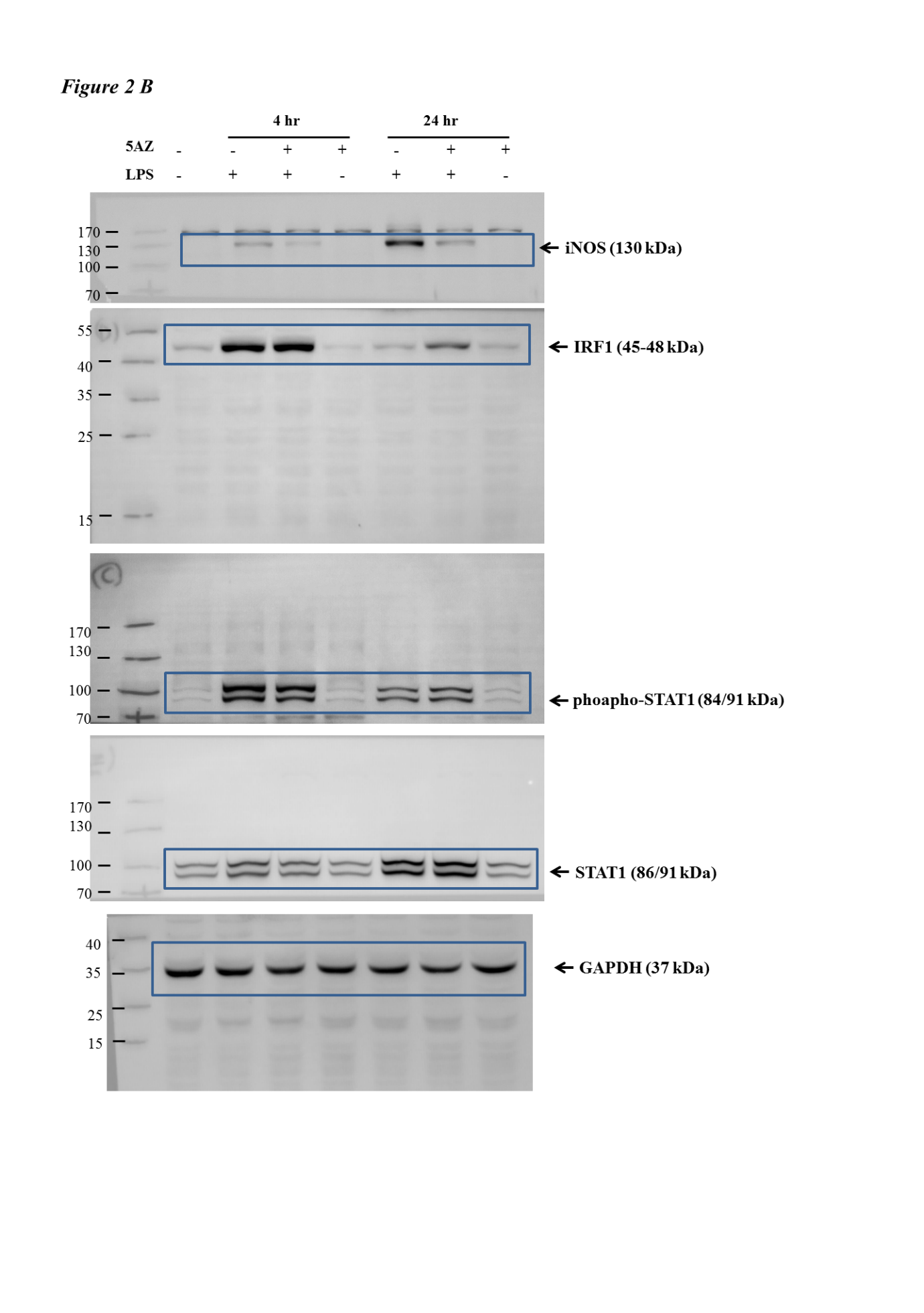
**

**
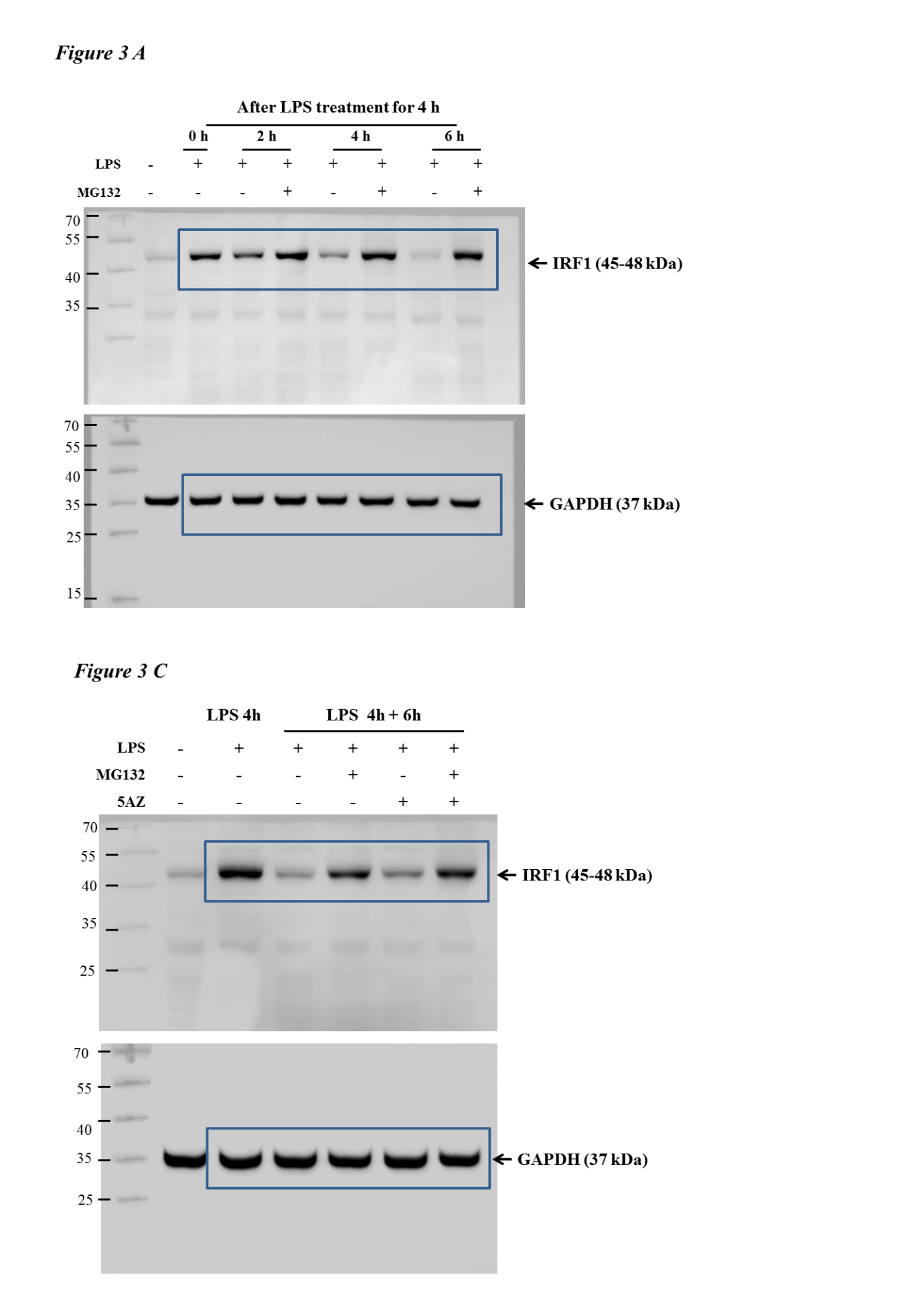
**

**
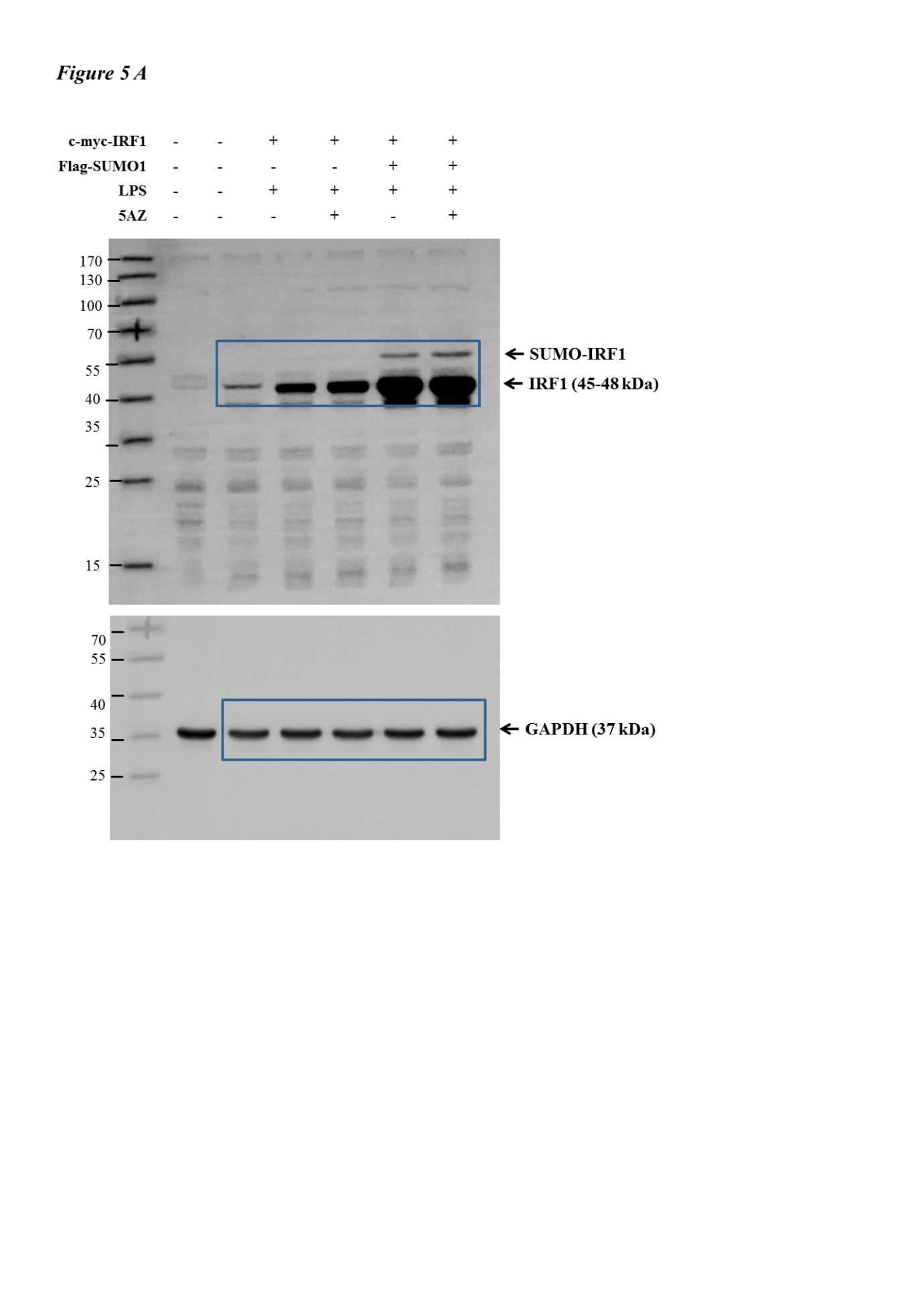
**

**
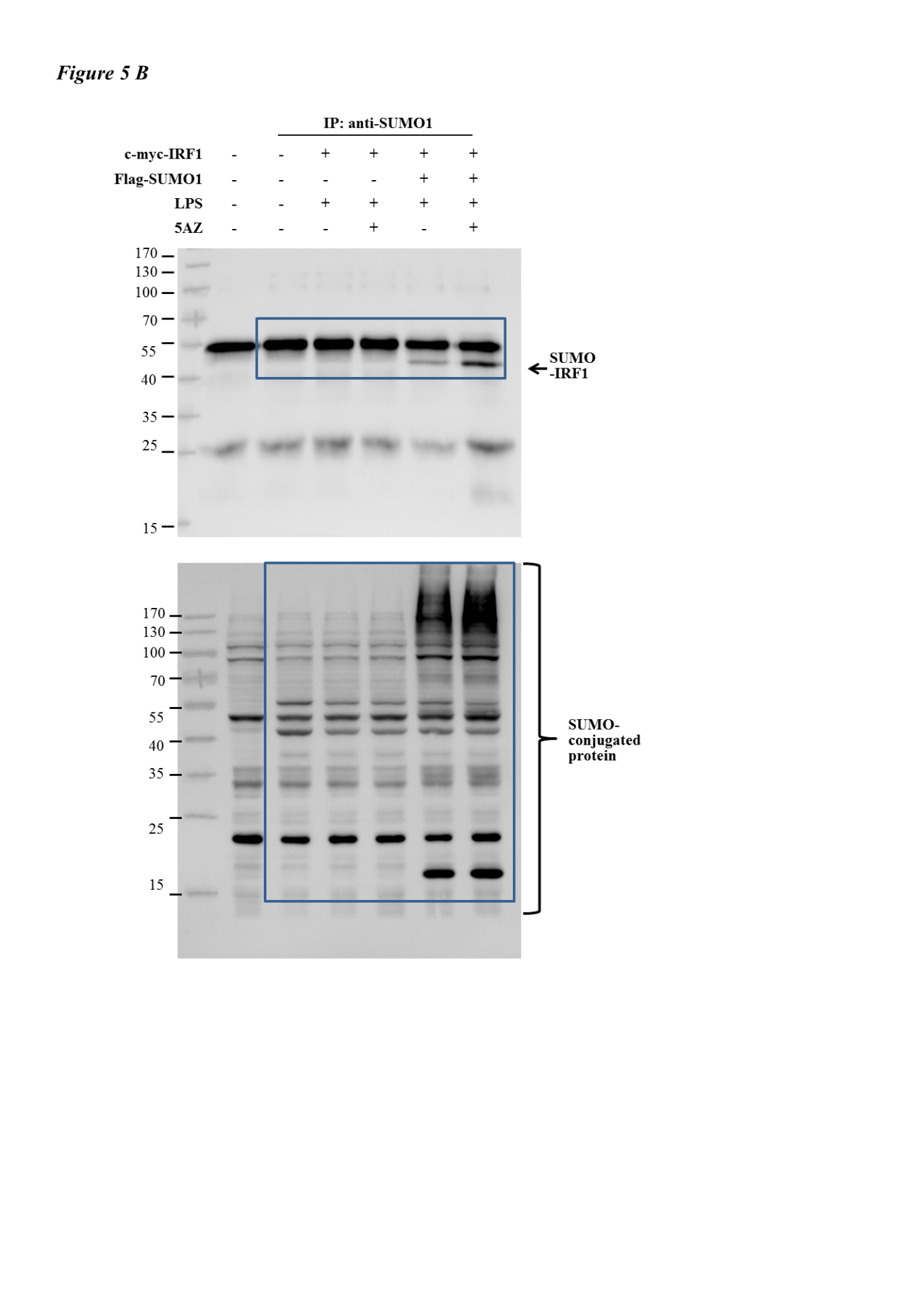
**

**
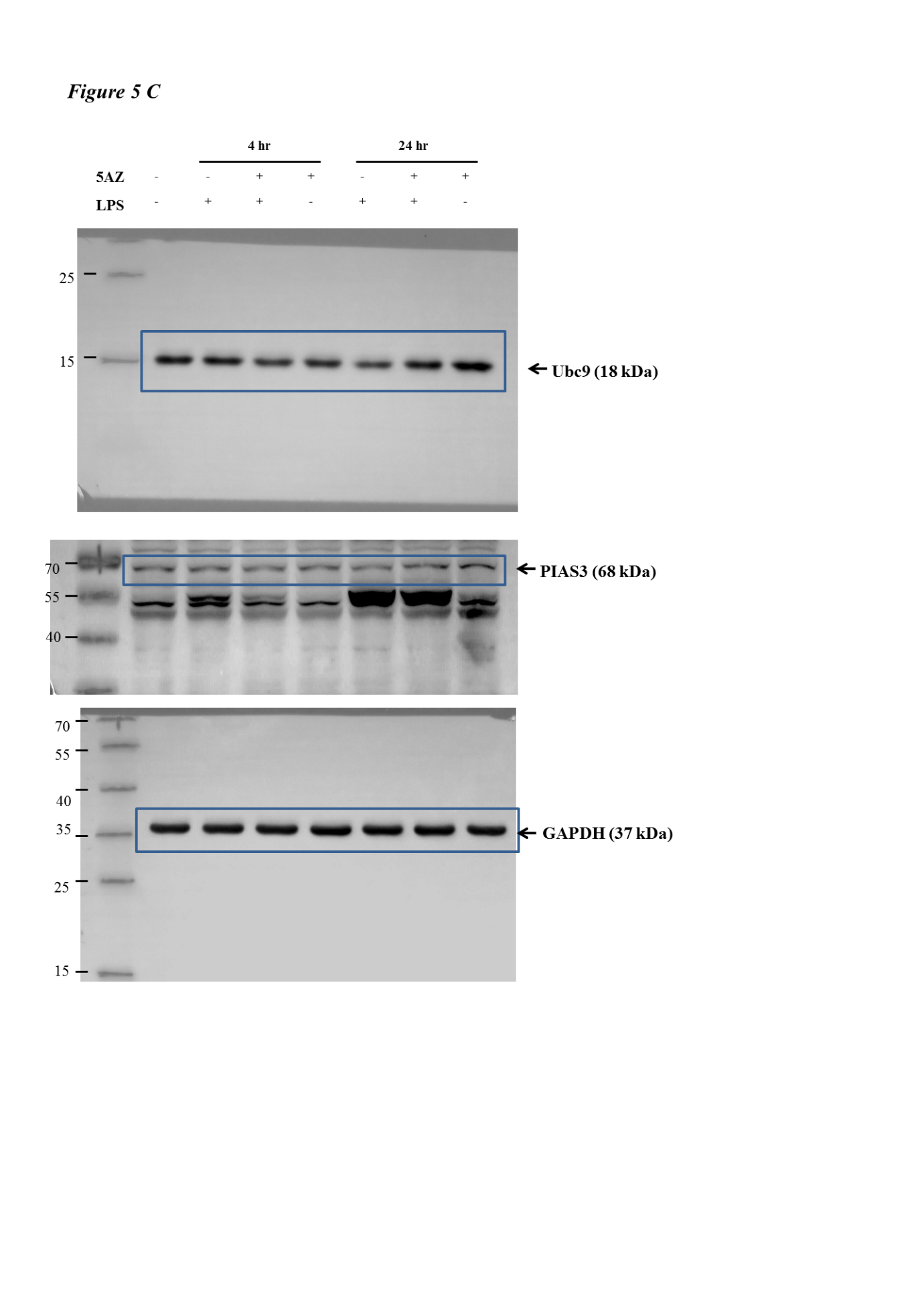
**

**
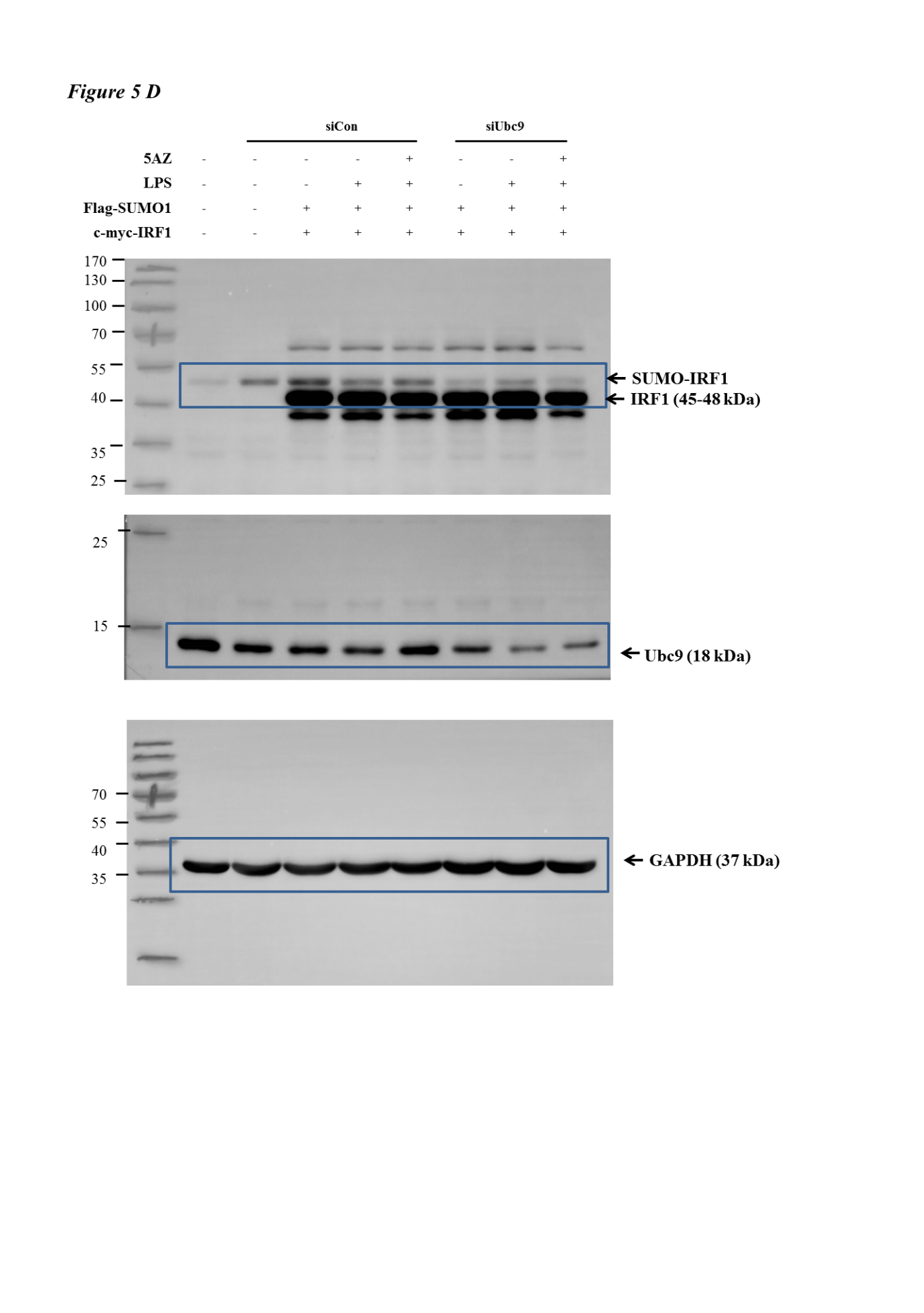
**

**
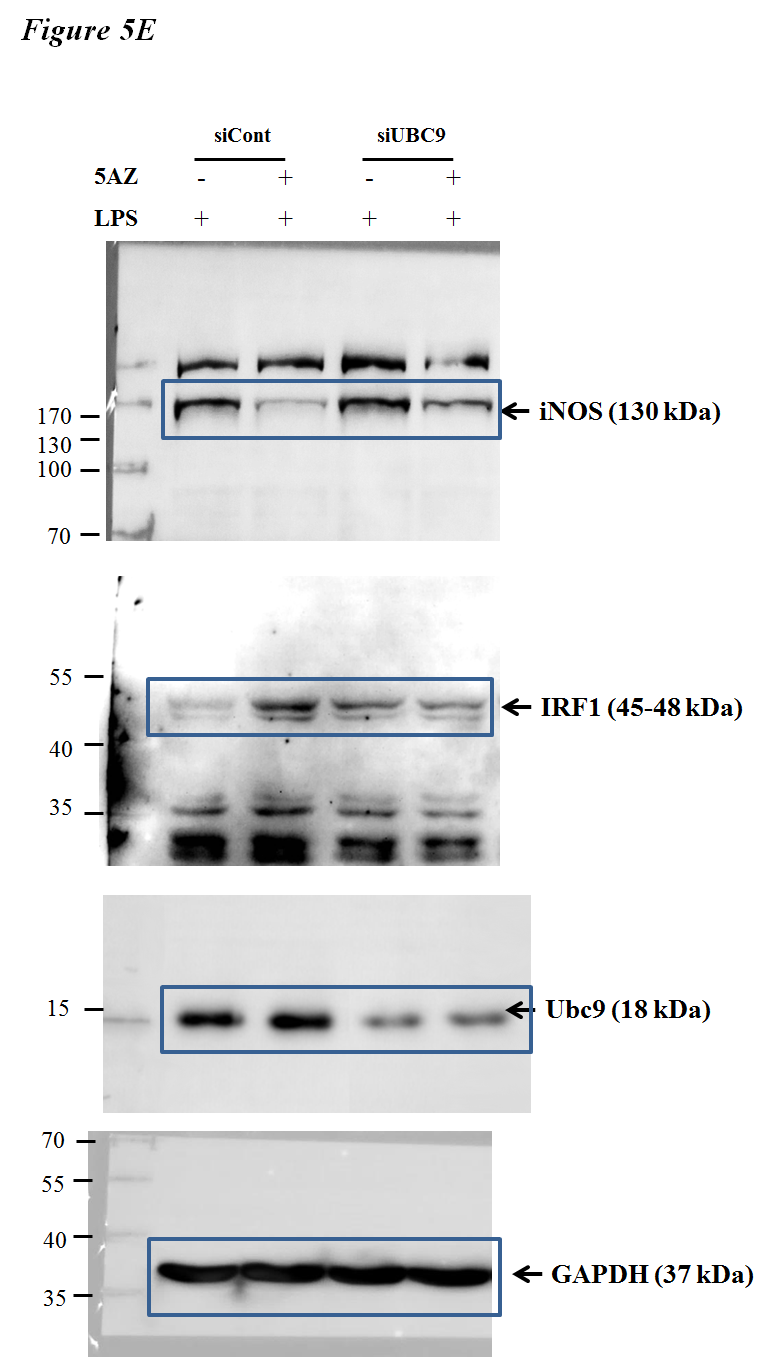
**

**Table S1. PCR primer sequences**

| Gene symbol | Forward primer | Reverse primer |
| --- | --- | --- |
| *NOS2* | TCACCTTCGAGGGCAGCCGA | TCCGTGGCAAAGCGAGCCAG |
| *IRF1* | TTAGCCCGGACACTTTCTCTGATGG | GTCCCCTCGAGGGCTGTCAATCTCT |
| *CD206* | CTGCAGATGGGTGGGTTATT | GGCATTGATGCTGCTGTTATG |
| *IL4R* | CTAGCTCCGTGCCCTTATTTAC | GGTTGGCTTCTGGTGGTATT |
| *GAPDH* | TGTGATGGGTGTGAACCACG | CAGTGAGCTTCCCGTTCAGC |
